# Comprehensive Analysis of the Tumor Immune Microenvironment Landscape in Glioblastoma Reveals Tumor Heterogeneity and Implications for Prognosis and Immunotherapy

**DOI:** 10.3389/fimmu.2022.820673

**Published:** 2022-03-02

**Authors:** Rongrong Zhao, Ziwen Pan, Boyan Li, Shulin Zhao, Shouji Zhang, Yanhua Qi, Jiawei Qiu, Zijie Gao, Yang Fan, Qindong Guo, Wei Qiu, Shaobo Wang, Qingtong Wang, Ping Zhang, Xing Guo, Lin Deng, Hao Xue, Gang Li

**Affiliations:** ^1^Department of Neurosurgery, Qilu Hospital, Cheeloo College of Medicine and Institute of Brain and Brain-Inspired Science, Shandong University, Jinan, China; ^2^Shandong Key Laboratory of Brain Function Remodeling, Qilu Hospital, Shandong University, Jinan, China

**Keywords:** glioblastoma, tumor immune microenvironment, proteomics, metabolomics, immunotherapy, target therapy

## Abstract

**Background:**

Glioblastoma (GBM) is a fatal brain tumor with no effective treatment. The specific GBM tumor immune microenvironment (TIME) may contribute to resistance to immunotherapy, a tumor therapy with great potential. Thus, an in-depth understanding of the characteristics of tumor-infiltrating immune cells is essential for exploring biomarkers in GBM pathogenesis and immunotherapy.

**Methods:**

We estimated the relative abundances of 25 immune cell types in 796 GBM samples using single sample gene set enrichment analysis (ssGSEA). Unsupervised clustering was used to identify different GBM-associated TIME immune cell infiltration (GTMEI) patterns. The GTMEIscore system was constructed with principal component analysis (PCA) to determine the immune infiltration pattern of individual tumors.

**Results:**

We revealed three distinct GTMEI patterns with different clinical outcomes and modulated biological pathways. We developed a scoring system (GTMEIscore) to determine the immune infiltration pattern of individual tumors. We comprehensively analyzed the genomic characteristics, molecular subtypes and clinicopathological features as well as proteomic, phosphoproteomic, acetylomic, lipidomic and metabolomic properties associated with the GTMEIscore and revealed many novel dysregulated pathways and precise targets in GBM. Moreover, the GTMEIscore accurately quantified the immune status of many other cancer types. Clinically, the GTMEIscore was found to have significant potential therapeutic value for chemotherapy/radiotherapy, immune checkpoint inhibitor (ICI) therapy and targeted therapy.

**Conclusions:**

For the first time, we employed a multilevel and multiplatform strategy to construct a multidimensional molecular map of tumors with different immune infiltration patterns. These results may provide theoretical basises for identifying more effective predictive biomarkers and developing more effective drug combination strategies or novel immunotherapeutic agents for GBM.

## Introduction

Glioblastomas (GBMs) are the most common aggressive primary brain tumors and the most lethal central nervous system (CNS) tumors due to their high proliferation rate, high aggressiveness, highly heterogeneous immunosuppressive microenvironment, and resistance to chemotherapy and targeted therapies (1, 2). Recently, immune checkpoint blockade (ICB) therapy has made outstanding achievements in improving the treatment of certain types of tumors. However, the unique immune microenvironment of the brain makes immunotherapy for GBM more challenging than that for other cancers (3). The tumor microenvironment (TME) is a key mediator of tumor malignant progression, plays an important role in clinical survival and response to therapy. In the TME, immune cells infiltrating into tumor tissue form the tumor immune microenvironment (TIME), which helps tumor cells achieve immune escape and promote tumor malignancy and is closely related to the response rate of immunotherapy (4, 5). Therefore, characteristics of the GBM immune microenvironment are expected to serve as biomarkers to guide clinical treatment and to identify GBM patients who can benefit from immunotherapy.

Mounting evidence suggests that cancer patients who receive personalized therapy show better clinical outcomes, and precision medicine promises to revolutionize universal therapy for oncology, but numerous studies still focus on abnormal changes in the genome (6, 7). As the understanding of tumor mechanisms deepens, the focus on tumor treatment is gradually shifting from tumor cells to the interaction between the tumor and the surrounding tissues. The TME is a key mediator of tumor progression and therapeutic outcome. Tumor cells are able to escape surveillance and recognition of the immune system and the killing of T cells without the combined effect of the immune microenvironment (8–11). The present classification schemes used for the TIME and the establishment of immune scoring systems in multiple tumors have greatly improved the current understanding of TIME subtypes (12–15). Strategies to further identify the ideal population and optimize immune combination strategies for specific populations are urgently needed in the era of precision medicine, and such strategies are popular areas of research in the field of immunotherapy at present. However, the recent integrated genomic and transcriptomic analyses and overall assessments of the GBM immune microenvironment have often been unsystematic, and effective immune models are lacking.

In this study, we integrated transcriptome information from 796 GBM samples, used single sample gene-set enrichment analysis (ssGSEA) to estimate the relative abundances of 25 immune cell types based on annotated immune cell gene expression profiles (16–18), and provided a comprehensive outlook on the immune landscape within GBM tumors. We revealed three distinct GTMEI patterns with different clinical outcomes and modulated biological pathways. In addition, we developed a scoring system to quantify the immune infiltration pattern of individual tumors, termed GTMEIscore. To understand the intrinsic tumor characteristics and tumor immune infiltration patterns associated with the GTMEIscore, we comprehensively analyzed the genomic characteristics, molecular subtypes and clinicopathological features as well as proteomic, phosphoproteomic, acetylomic, lipidomic and metabolomic properties associated with the GTMEIscore, revealing lots of novel dysregulated pathways and precise targets in GBM. Moreover, GTMEIscore accurately quantified the immune status of many other cancer types. Clinically, GTMEIscore was found to have significant potential therapeutic value for chemotherapy/radiotherapy, immune checkpoint inhibitor (ICI) therapy and targeted therapy. These findings might provide a theoretical basis for identifying more effective GBM predictive biomarkers and developing more effective, targeted clinical treatment strategies, ultimately guiding GBM clinical treatment and achieving precision medicine.

## Methods

### Collection and Preprocessing of Publicly Available Expression Datasets

A total of 796 GBM patients with clinical prognostic information from 6 cohorts, including 153 from the TCGA RNA-seq dataset, 374 from 2 CGGA RNA-seq datasets (237 from one cohort and 137 from the other cohort), 155 from the Gravendeel microarray dataset, 97 from the Wang RNA-seq dataset (19) and 17 patients treated with anti-PD1 therapy from the Zhao/PD1 RNA-seq dataset (20), were included in this study. TCGA RNA-seq data (FPKM format) and clinical information were downloaded from the TCGA database (https://portal.gdc.cancer.gov/repository) and transformed into TPM format. The 2 CGGA RNA-seq datasets and their clinical information were downloaded from the CGGA database (http://www.cgga.org.cn/), the Gravendeel microarray dataset and clinical information were downloaded from the GlioVis database (http://gliovis.bioinfo.cnio.es/), the Wang RNA-seq dataset (FPKM format) and clinical information were extracted from the supplemental data of the article, and the missing data were obtained with the K-nearest neighbor (KNN) method and transformed into TPM format. For the Zhao/PD1 RNA-seq dataset, we downloaded the raw data from SRA PRJNA482620 and then processed them into TPM format, and clinical information was obtained from the supplemental data of the article. The ComBat method from the ‘SVA’ R package was used to remove the batch effects among these different datasets (21). The basic information of all enrolled datasets is summarized in **Supplementary Table S1**.

For ICB data, RNA-seq data and clinical information from the IMvigor210 cohort, were obtained from http://research-pub.Gene.com/imvigor210corebiologies based on the Creative Commons Attribution 3.0 license. The metastatic melanoma RNA-seq data from patients treated with nivolumab were obtained from GSE78220 (22) and GSE91061 (23) datasets, and the clinical information were obtained from the supplemental data of the article, respectively.

### Estimation of TME Immune Cell Infiltration

We used the ssGSEA algorithm to quantify the relative abundances of 25 infiltrating immune cells in the GBM TME. Gene sets for BMDM TAMs and MG TAMs were obtained from Bowman, R. et al. (18), and those for 23 other infiltrating immune cell types were obtained from Charoentong P. et al. (24, 25). The relative abundance of each infiltrating immune cell in each sample was represented by the enrichment score calculated by the ssGSEA algorithm. The ESTIMATE algorithm was used to assess the immune and stromal scores and tumor purity of each GBM sample.

### Statistical Analysis

Spearman and distance correlations were used to calculate the correlation coefficient of 25 immune cell types. Student’s t-test was used for two-group comparisons. For comparisons among more than two groups, the Kruskal-Wallis test and one-way ANOVA were used for nonparametric and parametric data. The cutoff values of each dataset (other than the TCGA dataset) were evaluated based on the association between survival time and the GTMEIscore using the “survminer” package, and the TCGA dataset was grouped according to the median GTMEIscore. The Kaplan-Meier method was used to generate survival curves for the subgroups in each dataset, and the log-rank (Mantel-Cox) test was used to determine if they were significantly different. The hazard ratios (HRs) for the univariate analyses were calculated using a univariate Cox proportional hazards regression model. Univariate prognostic analysis results were visualized using the “forestplot” R package. The specificity and sensitivity of the GTMEIscore in predicting response to anti-PD1 therapy were assessed by receiver operating characteristic (ROC) curves, and the area under the curve (AUC) was quantified using the “pROC” R package. P>0.05 was considered to indicate nonsignificance (ns), and P<0.05 was considered to indicate statistical significance (*P<0.05; **P <0.01; ***P<0.001, ****P<0.0001). All data processing with R packages was performed using R Studio (version 3.6.3).

## Results

### Landscape of Immune Cell Infiltration and Clinicopathological Characteristics of TME Subtypes in GBM

**Figure 1:**
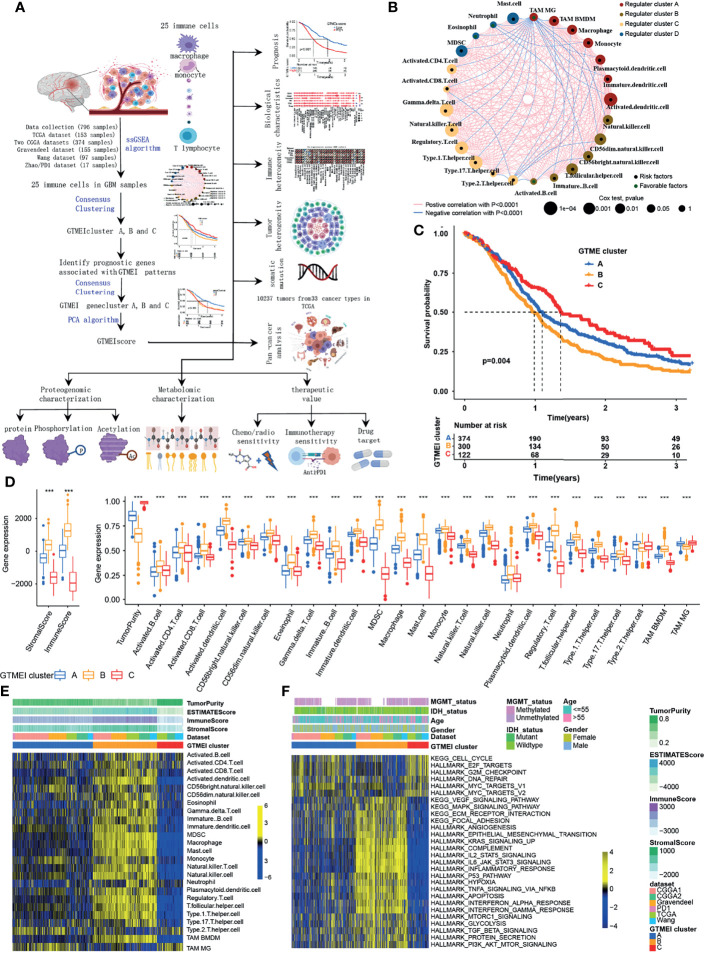
Landscape of immune cell infiltration and clinicopathological characteristics of TME subtypes in GBM. **(A)** Overview of the workflow of our research. **(B)** Cellular interactions of 25 immune cell types in the GBM microenvironment. The size of each immune cell represents its effect on the survival of GBM patients, as calculated using a log10 formula (log-rank test P value). Green indicates that the immune cell is a protective factor for overall survival (OS), while black indicates a risk factor. The lines connecting the immune cells indicate cell-cell interactions, the thickness of which indicates the strength of the correlation estimated by Spearman correlation analysis. Positive correlations are indicated in red, and negative correlations are indicated in blue. **(C)** Kaplan-Meier curves for the OS of 796 GBM patients from 6 GBM cohorts with three GTMEI clusters. The numbers of patients in GTMEI clusters A, B and C were 374, 300 and 122, respectively, and the log-rank test showed P = 0.004. **(D)** Abundances of immune/stromal score, tumorpurity and 25 immune cell types in the three GTMEI patterns. The upper and lower ends of the boxes indicate the interquartile range of the values. The lines in the boxes represent the median values, and black dots show outliers. The significance of differences between the three clusters were determined by the Kruskal-Wallis test. *P < 0.05; **P < 0.01; ***P < 0.001. **(E)** Unsupervised clustering of 25 immune cell types in the cohort of 796 GBM patients. A heatmap was used to visualize immune cell infiltration. Yellow represents high immune cell abundance, black represents moderate immune cell abundance, and blue represents low immune cell abundance. **(F)** GSVA revealed the activation status of biological pathways in different GTMEI patterns. A heatmap was used to visualize these biological pathways. Yellow represents activated pathways, and blue represents inhibited pathways.

The overview workflow of our research is shown in **Figure 1A**. First, we combined six GBM datasets (a TCGA dataset, two CGGA datasets, The Gravendeel dataset, the Wang dataset and the Zhao/PD1 dataset) with available survival data and clinical information into one meta-cohort, including 796 samples (**Supplementary Table S1**). We then performed ssGSEA and employed the ESTIMATE algorithm to quantify the abundances of immune cells in and the immune and stromal scores of GBM tumor tissues (**Supplementary Table S1**) and depicted a comprehensive landscape of TME immune cell interactions, regulatory connections and their prognostic value for patients with GBM (**Figure 1A** and **Supplementary Table S2**). We identified three independent GTMEI clusters with significant survival differences (**Figure 1B**, P = 0.004; **Supplementary Figure S1A** and **Table S1**). We then explored the specific differences in the abundances of immune/stromal score, tumorpurity and 25 TME-infiltrating immune cells and their intrinsic biological differences among GTMEI clusters. We found that GTMEI cluster B, which had the worst prognosis, presented significantly increased immune cell infiltration and immune and stromal scores and enrichment of both immune and stromal activation-related pathways; GTMEI cluster A, which showed survival times between those of GTMEI cluster B and GTMEI cluster C and was characterized by moderate immune cell infiltration, was prominently associated with activation of carcinogenic and stromal pathways; However, GTMEI cluster C, which was associated with a favorable prognosis and was characterized by suppression of immunity, was prominently associated with activation of the cell cycle and DNA repair pathways (**Figures 1C–F**).

### Generation of the GTMEI Gene Signature and Functional Annotation

**Figure 2:**
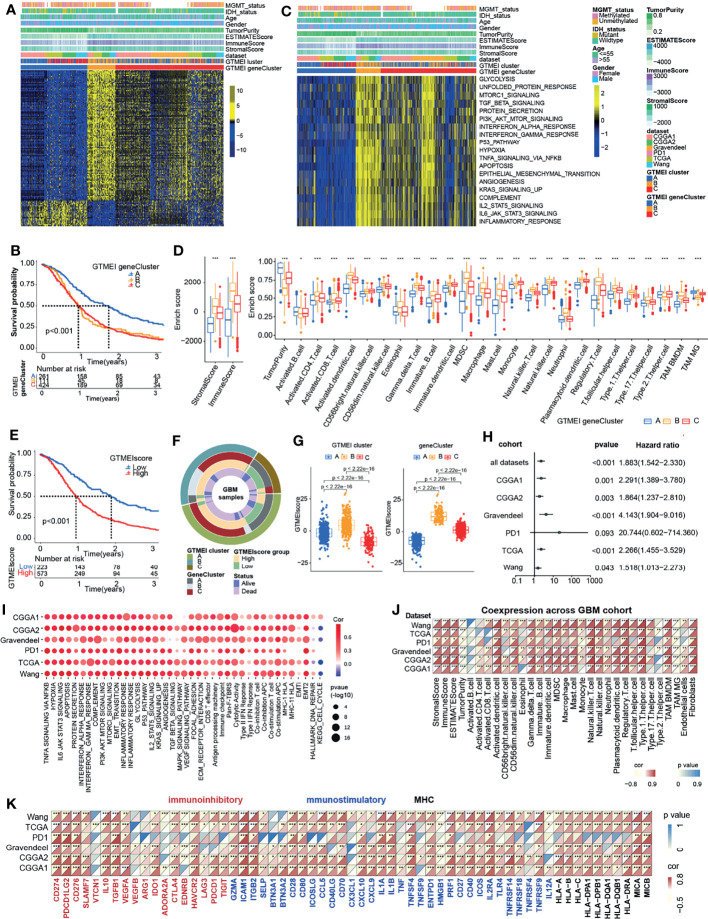
Generation of the GTMEI gene signature and functional annotation. **(A)** Based on the expression data of 796 GBM patients, unsupervised clustering of representative DEGs associated with the GTMEI patterns was performed to classify patients into three groups, called GTMEI gene clusters A-C. A heatmap was used to visualize the gene expression. Yellow represents high expression, black represents moderate expression, and blue represents low expression. **(B)** Kaplan-Meier curves for the OS of 796 GBM patients within the three GTMEI gene clusters. The numbers of patients in GTMEI gene clusters **(A–C)** were 261, 111 and 424, respectively, and the log-rank test showed P < 0.001. C GSVA revealed the activation status of biological pathways in different GTMEI gene clusters. A heatmap was used to visualize these biological pathways. Yellow represents activated pathways, and blue represents inhibited pathways. **(D)** Abundances of immune/stromal score, tumorpurity and 25 immune cell types in the three GTMEI gene clusters. The upper and lower ends of the boxes indicate the interquartile range of values. The lines in the boxes represent the median values, and black dots show outliers. The significance of differences between the three clusters were assessed by the Kruskal-Wallis test. *P < 0.05; **P < 0.01; ***P < 0.001. **(E)** Kaplan-Meier curves for the OS of 796 GBM with a high GTMEIscore (n = 573) and a low GTMEIscore (n = 223), and the log-rank test showed P < 0.001. **(F)** CircGroup plot showing the relationships between GTMEI clusters, GTMEI gene clusters, the GTMEIscore, and survival status. **(G)** Differences in the GTMEIscore among the three (left) GTMEI clusters and (right) GTMEI gene clusters in 796 GBM patients. **(H)** Univariate Cox analysis of the prognostic value of the GTMEIscore for survival in the combined GBM cohort as well as in the independent GBM cohorts. A hazard ratio (HR) > 1.0 indicated that a high GTMEIscore was an adverse prognostic biomarker. **(I)** Spearman correlation analysis of the GTMEIscore and classical signaling pathways in independent GBM cohorts. Blue indicates negative correlations, and red indicates positive correlations. The size of the circle represents the statistical P value, with larger circles representing greater statistical significance. **(J)** Spearman analysis of the correlation of the GTMEIscore with **(J)** the abundances of 25 immune cells and **(K)** immunomodulators (immunoinhibitory, immunostimulatory and MHC molecules). Colors indicate correlation coefficients, with yellow indicating a negative correlation and red indicating a positive correlation. Asterisks indicate statistically significant P values calculated using Spearman correlation analysis. *P < 0.05; **P < 0.01; ***P < 0.001.

To further investigate the underlying genetic alterations and biological behavior of each GTMEI pattern, we identified 2288 GTMEI pattern phenotype-related DEGs using the “limma” package (**Supplementary Figure S1B** and **Table S3**). Further enrichment analysis of the DEGs *via* the Metascape database showed that they were significantly involved in the cell cycle, DNA repair and immune-related pathways (**Supplementary Figure S1C** and **Table S3**). Furthermore, enrichment analysis *via* PaGenBase showed that these genes were almost exclusively expressed in the blood, spleen, bone marrow, and thymus and some other tissues where peripheral immune cells gather (**Supplementary Figure S1D**). We further used the random forest algorithm for 2288 GTMEI phenotype-related DEGs to dimensionality reduction, and then extracted 135 genes with significant prognostic value (P<0.001) as the most representative GTMEI pattern DEGs (we call them as GTMEI phenotype signature), which were significantly enriched in immune and metabolism-related signaling pathways (**Supplementary Figure S1E** and **Supplementary Table S4**). Consistent with the clustering groups of GTMEI patterns, unsupervised hierarchical clustering analysis also identified three distinct GTMEI pattern-related genomic phenotypes based on the expression of the GTMEI phenotype signature, and we named these three clusters as GTMEI gene clusters A-C (**Figure 2A** and **Supplementary Figures S1F, G**). Furthermore, we found that patients in gene cluster A, who showed immune suppression, had a better prognosis, whereas patients in gene cluster B, who showed high immune cell infiltration and activation of immune, stromal and carcinogenic pathways, had the most unfavorable outcomes (**Figures 2B–D**), which was consistent with the expected outcomes of the GTMEI patterns.

Given the heterogeneity and complexity of the GTMEI patterns, we constructed a DEG-based scoring system, termed the GTMEI phenotype score (GTMEIscore), based on the GTMEI phenotype signature to quantify the GTMEI pattern of individual patients using PCA. The patients were grouped into high or low GTMEIscore groups using the cutoff value obtained with the “survminer” package, and patients with a low GTMEIscore exhibited a significant survival benefit (**Figure 2E**, P < 0.001). And GTMEI gene clusters B and C were linked to a higher GTMEIscore, whereas GTMEI gene cluster A exhibited a lower GTMEIscore (**Figure 2F**). Further analysis showed that both GTMEI cluster B and gene cluster B had the highest GTMEIscore (**Figure 2G**). We then tested whether the GTMEIscore could serve as an independent prognostic biomarker for GBM patients. As shown in **Figure 2H**, the robust prognostic value of the GTMEIscore was validated in six independent datasets. To further evaluate the biological relevance of the GTMEIscore system, we explored the correlation of the GTMEIscore with immune-related pathways as well as known carcinogenic signatures and found that it was positively correlated with the immune activation process, oncogenic activation and stromal activation signaling but negatively correlated with the cell cycle and DNA repair process (**Figure 2I**). A heatmap of the correlation matrix demonstrated that the GTMEIscore was markedly positively correlated with the immune and stromal scores but negatively correlated with tumor purity. Regarding immune cells, the GTMEIscore was positively correlated with most infiltrating immune cells, as well as with fibroblasts (**Figure 2J**). The expression levels of most MHC, immunostimulatory, and immunoinhibitory molecules were also positively associated with the GTMEIscore (**Figure 2K**). These results implied that the GTMEIscore can reflect immune cell infiltration and can be a reliable prognostic biomarker.

### Molecular Subtypes and Tumor Somatic Mutations Associated With and Chemotherapy/Radiotherapy Prognostic Value of the GTMEIscore

**Figure 3:**
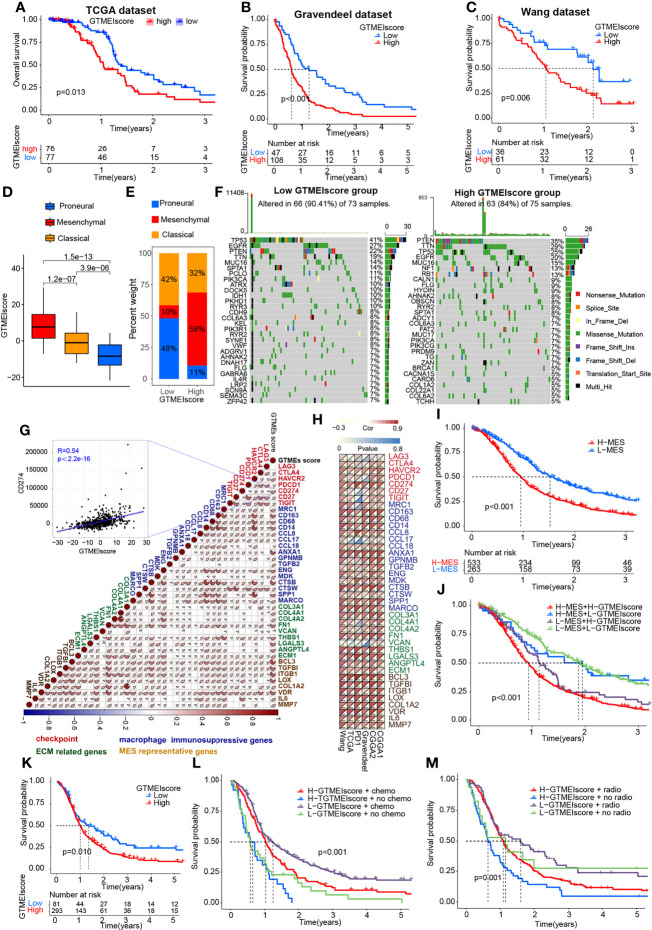
Molecular subtypes and tumor somatic mutations associated with and the chemotherapy/radiotherapy prognostic value of the GTMEIscore. Kaplan-Meier curves showing OS for the high (red) and low (blue) GTMEIscore groups in the **(A)** TCGA dataset, **(B)** Gravendeel dataset, and **(C)** Wang dataset, and the log-rank test showed P = 0.013, P<0.001 and P=0.006, respectively. **(D)** Differences in GTMEIscore among different GBM molecular subtypes. The Kruskal-Wallis test was used to determine the significance of differences between the three GBM molecular subtypes. **(E)** Stacked bar plot of GBM molecular subtypes in the high and low GTMEIscore groups. **(F)** Waterfall plot of the tumor somatic mutation landscape in the low GTMEIscore (left) and high GTMEIscore (right) groups. Each bar represents the mutation information for an individual patient. The top bar plot shows TMB, and the numbers on the right indicate the mutation frequency of each gene. The bar plot on the right shows the proportion of each mutation type. **(G)** Spearman analysis was used to determine the correlation of the GTMEIscore with the expression of immunosuppression-related genes (checkpoint molecule genes, macrophage immunosuppressive genes, ECM-related genes and MES representative genes) in the combined GBM dataset. Colors indicate correlation coefficients, with blue indicating a negative correlation and red indicating a positive correlation. The size of the sector represents the correlation coefficient, and a larger angle means a stronger correlation. **(H)** Spearman analysis of the correlation of the GTMEIscore with the expression of immunosuppression-related genes molecules (checkpoint molecule genes, macrophage immunosuppressive genes, ECM-related genes and MES representative genes) in six independent GBM datasets. **(I)** Kaplan-Meier curves for the OS of 796 GBM patients in the high MES score (n = 533) and low MES score (n = 263) groups, and the log-rank test showed P < 0.001. **(J)** Survival analysis was performed using Kaplan-Meier curves for the subgroup of patients stratified according to GTMEIscore combined with MES score, and the log-rank test showed P < 0.001. H: High; L: Low. **(K)** Kaplan-Meier curves for the OS of the high GTMEIscore (n = 293) and low GTMEIscore (n=81) groups in 2 CGGA GBM datasets, and the log-rank test showed P = 0.010. Survival analysis was performed using Kaplan-Meier curves for subgroups of patients stratified by GTMEIscore **(L)** and treatment with adjuvant chemotherapy (chemo) (the log-rank test showed P < 0.001) by GTMEIscore **(M)** and treatment with adjuvant radiotherapy (radio) (the log-rank test showed P = 0.001).

To better understand the determinants of GBM tumor evolution and treatment resistance, we then evaluated the differences in GTMEIscore among TCGA molecular subtypes in the TCGA, Gravendeel and Wang datasets, in which clinical information was available. Survival analysis showed that the high GTMEIscore group in the TCGA dataset had shorter survival (**Figure 3A**, P = 0.013), which was further validated in the Gravendeel dataset (**Figure 3B**, P = 0.006) and Wang dataset (**Figure 3C**, P = 0.006). Samples with a higher GTMEIscore were clearly concentrated in the MES subtype, which had a poor prognosis (**Figure 3D**), and samples with the MES subtype were also mainly concentrated in the high GTMEIscore group in the TCGA dataset (**Figure 3E**); the same patterns were observed in the Gravendeel and Wang datasets (**Supplementary Figures 2A–D**).

We then analyzed the differences in the distribution of somatic mutations between the high and low GTMEIscore groups in the TCGA-GBM cohort and Wang cohort using the “maftools” package and found that the PTEN mutation rate (low: 22%, high: 35%) was significantly increased in the high GTMEIscore group compared to the low GTMEIscore group (**Figure 3F** and **Supplementary Figure 2E**). Chen et al. (26) found that PTEN deficiency in GBM increases macrophage infiltration, and the infiltrated macrophages in turn secrete SPP1 to support GBM survival *via* activating YAP1 signaling. Moreover, NF1 mutation, a MES subtype marker that drives recruitment of TAMs (27), was also remarkably more prevalent in the high GTMEIscore group (13%) than in the low GTMEIscore group (5%). In our study, we showed that there was a significant positive correlation between the GTMEIscore and macrophages, especially BMDM TAMs (hereafter also called macrophages), indicating the presence of a large group of infiltrating mononuclear-derived macrophages in the tumor tissues of GBM samples with a high GTMEIscore (**Figure 2J**). We also found a significant positive correlation between the GTMEIscore and the expression of myeloid cell-derived macrophage-restricted chemokines, representative MES genes, genes encoding ECM and immune checkpoint molecules in both the combined and independent datasets (**Figures 3G, H**). In addition, GSEA also showed that YAP1 signaling (CORDENONSI_YAP_CONSERVED_SIGNATURE) and MES signature was significantly upregulated in the high GTMEIscore group compared to the low GTMEIscore group (**Supplementary Figures S2F, G**). We next calculated the enrichment scores of individual GBM samples according to the MES gene signature (27) and found that the high MES expression group had a significantly worse prognosis than the low MES expression group (**Figure 3I**, P < 0.001). Further analysis revealed a significant survival advantage for patients with both a low GTMEIscore and low MES score (**Figure 3J**, P < 0.001). Next, we explored the relationship between the GTMEIscore and GBM chemotherapy/radiotherapy sensitivity in the CGGA dataset, for which chemotherapy/radiotherapy data were available. We further demonstrated that the prognosis of the group with a high GTMEIscore was significantly worse than that of the low GTMEIscore group (**Figure 3K**, P = 0.010). Further analysis showed that patients in the low GTMEIscore group and those treated with chemotherapy/radiotherapy had a significant survival advantage, while patients in the high GTMEIscore group and those not treated with chemotherapy/radiotherapy had the worst prognosis (**Figures 3L, M**).

### Correlation Between the GTMEIscore and Proteomic Characteristics

**Figure 4:**
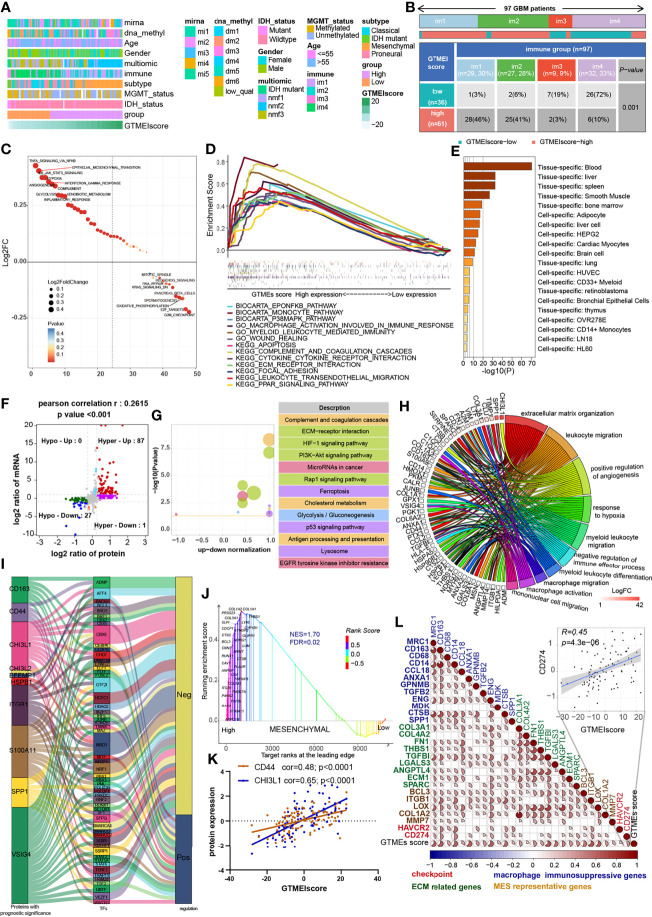
Correlation between the GTMEIscore and proteomic characteristics. **(A)** Heatmap describing the relationship between the GTMEIscore and various clinical features in the Wang cohort. miRNA, DNA methylation, multiomics, immune cell molecular, TCGA molecular subtype, age, sex, MGMT and IDH mutation status annotations are provided as examples. **(B)** Heatmap and table of the distribution of GBM immune subtypes (im1, im2, im3 and im4) between the high and low GTMEIscore groups, chi-square test showed P = 0.001. **(C)** GSVA showing differences in hallmark biological pathways between the high and low GTMEIscore groups. Scatter plots were used to visualize these differences in pathways. The size of the circle indicates the size of the fold change (FC), and the color indicates the statistical significance of the difference. The color red indicates statistical significance, and blue indicates statistical insignificance. **(D)** GSEA showing the gene sets enriched in high GTMEIscore subgroup (P < 0.05, FDR < 0.25). **(E)** Summary enriched genes positively correlated with the GTMEIscore at the protein level in the PaGenBase database. **(F)** Dot plot of log2FC (mRNA expression) versus log2FC (protein expression) values showing a positive correlation between the overall mRNA expression and protein expression levels (Pearson’s r = 0.2615; P < 0.001) and the distribution of genes with significant changes in both the mRNA (|FC| > 2, P < 0.05) and corresponding protein expression (|FC| > 1.2, P < 0.05) levels in the high GTMEIscore group compared with the low GTMEIscore group. Colored circles indicate significant changes in at least the mRNA or the protein expression of the gene. **(G)** KEGG enrichment analysis of 87 genes (Hyper-Up) and 27 genes (Hypo-Down) that were significantly differentially expressed at both the mRNA and protein levels. **(H)** GO BP enrichment analysis of 87 genes that were significantly upregulated at both the mRNA and protein levels. **(I)** Alluvial plot showing regulatory network relationships between proteins with prognostic significance and transcription factors (TFs). **(J)** GSEA of mesenchymal signatures showing that GBM samples from the high GTMEIscore group were enriched in the MES-subtype group compared to GBM samples from the low GTMEIscore group. NES, normalized enrichment score; FDR, false discovery rate. **(K)** Correlation scatter plot showing that the GTMEIscore was positively correlated with the expression of CD44 and CHI3L1, markers of the MES subtype. **(L)** Spearman analysis of the correlation between the GTMEIscore and the protein expression of immunosuppression-related genes (checkpoint molecule genes, macrophage immunosuppressive genes, ECM-related genes and MES representative genes). Colors indicate correlation coefficients, with blue indicating a negative correlation and red indicating a positive correlation. The size of the sector represents the correlation coefficient, and a larger angle means a stronger correlation.

Understanding the proteomic characteristics of the GTMEIscore can help us better understand the GBM TME pattern. By integrating proteomic and metabolomic data from up to 10 platforms, Wang et al. (19) identified new multi-omics subtypes. We then analyzed the relationship between the GTMEIscore and clinical features as well as the identified molecular subtypes and found that the high GTMEIscore group tended to have more nmf2-subtype samples (mesenchymal-like, which mainly showed enrichment of immune response and extracellular matrix organization pathways), MES-subtype samples, fewer IDH mutations, and worse pathological features (**Figure 4A**). In addition, we found that the high GTMEIscore group mainly showed enrichment of the im1 and im2 subtypes, which was characterized by high enrichment of immune cells (**Figure 4B**, P=0.001, chi-square test). To explore the underlying mechanisms that led to the different results in the low and high GTMEIscore groups, we annotated the protein data with the hallmark dataset and performed differential analysis; we found that pathways related to the cell cycle were enriched in the low GTMEIscore group, while pathways related to the immune response and ECM remodeling were enriched in the high GTMEIscore group, consistent with the RNA results (**Figure 4C**). Further GSEA also showed consistent results (**Figure 4D**). In addition, Metascape database (28) analysis revealed that genes with a significant positive association with the GTMEIscore (**Supplementary Table S5**, Pearson r > 0.3, P < 0.05) were significantly enriched in pathways related to the regulation of cell biological functions, stromal activation and immunity(**Supplementary Figure S3A** and **Table S6**). Further PaGenBase (29) enrichment analysis showed that these genes were mainly specifically expressed in peripheral immune organs such as the spleen, blood and bone marrow (**Figure 4E**). The coanalysis of proteomic and transcriptomic alterations helped us to further decipher the mechanism by which the TIME in GBM is formed. We analyzed the differences between the high and low GTMEIscore groups based on RNA-seq data and found that compared with the low GTMEIscore group, the high GTMEIscore group had 183 DEGs (FC>2 and P value<0.05) (**Supplementary Table S7**). For the protein data, we found 2758 DEGs (FC>1.2 and P value<0.05) in the high GTMEIscore group compared with the low GTMEIscore group (**Supplementary Table S8**). As shown in **Figure 4F** and **Supplementary Table S9**, *via* joint analysis of the protein and RNA-seq data, we found a moderately strong positive correlation (Pearson r = 0.2615, P value <0.001) between the differences in mRNA and protein expression levels, and all genes were divided into four main groups: 87 genes that were simultaneously upregulated (Hyper-Up), 27 genes that were simultaneously downregulated (Hypo-Down). KEGG functional enrichment analysis showed that the Hyper-Up genes were mainly enriched in some classical oncogenic pathways, metabolic pathways and immune response-related pathways (**Figure 4G**). Further enrichment analysis *via* the GO database showed that the DEGs were notably enriched in the hypoxia, immune cell migration, angiogenesis, matrix remodeling, and macrophage activation pathways (**Figure 4H**). We then performed a univariate Cox prognostic analysis of the proteins and identified 13 proteins with significant prognostic significance (**Supplementary Figure 3B**). We obtained the list of transcription factors (TFs) from the Cistrome database, and subsequently, we examined the coexpression relationships between these prognosis-related proteins and TFs. We used the criteria of Pearson |r|>0.5 and P < 0.05 to obtain the coexpressed genes. Finally, we visualized the coexpression network information using alluvial plots (**Figure 4I**). The results suggested that the expression of the proteins may be regulated by these TFs. We further analyzed the relationship between nmf2-subtype genes and the GTMEIscore. As shown in **Supplementary Figure 3C**, as the GTMEIscore increased, the protein expression levels of these genes also showed a gradual increase. Similar to results from the RNA-seq data, the results of GSEA of the protein data also showed that the MES signature was significantly enriched in the high GTMEIscore group (**Figure 4J**, FDR=0.02). Moreover, the GTMEIscore showed a remarkable positive correlation with the expression of the MES markers CD44 and CHI3L1 (YKL40) at the protein level (**Figure 4K**). Our analysis revealed that, consistent with the mRNA data, the GTMEIscore was significantly and positively correlated with the expression level of immune checkpoint proteins, myeloid cell-derived macrophage-restricted chemokines, and MES-representative and ECM-related proteins (**Figure 4L**). These results facilitate the identification of important proteins associated with the formation of GBM TME patterns.

### Correlation Between the GTMEIscore and Protein Phosphorylation and Acetylation

**Figure 5:**
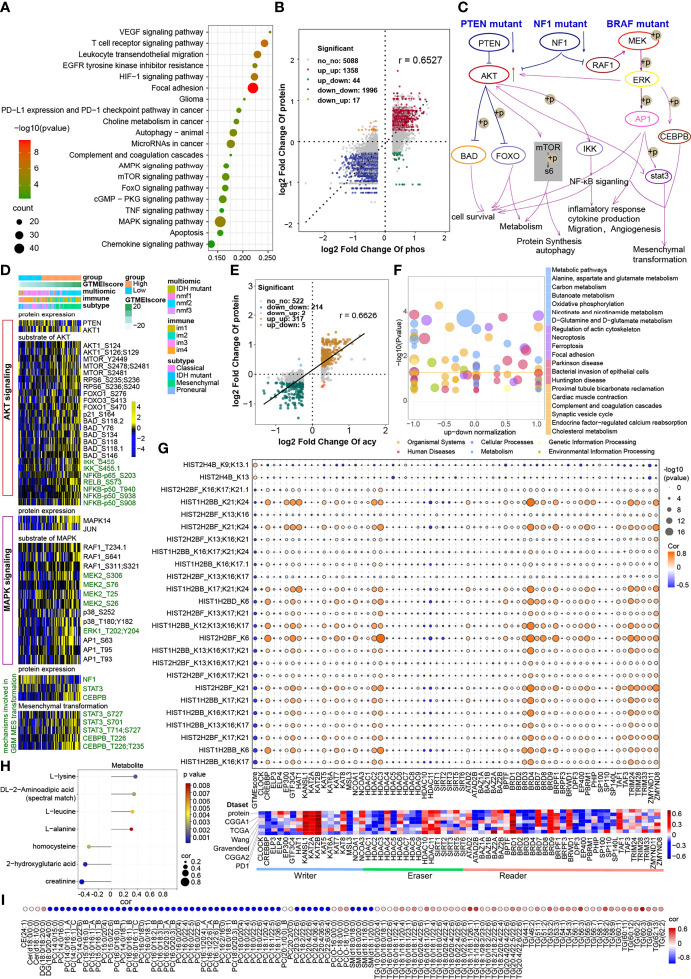
Correlation between the GTMEIscore and protein phosphorylation and acetylation. **(A)** KEGG enrichment analysis of proteins with upregulated phosphorylation levels. **(B)** Dot plot of log2 fold change (FC) (protein phosphorylation level) versus log2FC (protein expression) values showing a positive correlation between the overall protein phosphorylation level and protein expression level (Pearson’s r = 0.6527) and the distribution of genes with significant changes in both the phosphorylation level (|FC| > 1.2, P < 0.05) and corresponding protein expression (|FC| > 1.2, P < 0.05) in the high GTMEIscore group compared with the low GTMEIscore group. **(C)** Schematic diagram showing specific proteins and their downstream alterations based on somatic mutations. **(D)** Heatmap showing somatic mutation-based alterations in specific proteins and their downstream protein phosphorylation sites. **(E)** Dot plot of log2FC (protein acetylation level) versus log2FC (protein expression) values showing a positive correlation between the overall protein phosphorylation level and protein expression level (Pearson’s r = 0.6527) and the distribution of genes with significant changes in both the acetylation level (|FC| > 1.2, P < 0.05) and corresponding protein expression (|FC| > 1.2, P < 0.05) in the high GTMEIscore group compared with the low GTMEIscore group. **(F)** KEGG enrichment analysis of proteins with significantly altered acetylation levels (|FC| > 1.2, P < 0.05). **(G)** (Upper) Correlation of the GTMEIscore with histone acetylation sites and histone acetyltransferase, deacetylase, and reader levels. The size of the circle represents the significance, and the color represents the correlation coefficient. (Lower) Correlation of the GTMEIscore with histone acetyltransferase, deacetylase, and reader levels; the color represents the correlation coefficient. **(H)** Lollipop chart showing metabolites with a significant correlation with the GTMEIscore (Pearson r > 0.3, P value <0.05). I Metabolites with a significant correlation with the GTMEIscore (Pearson |r| > 0.3, P value < 0.05). **(I)** Bubble plots showing significant correlations of the GTMEIscore with lipids (Pearson |r| > 0.3, P value < 0.05).

The proteomic phosphorylation differential analysis data showed that 3811 phosphorylation sites of 1438 proteins were dramatically upregulated and 4873 phosphorylation sites of 1529 proteins were significantly downregulated in the high GTMEIscore group (**Supplementary Table S10**, FC > 1.2, P value < 0.05). KEGG enrichment analysis showed that proteins with upregulated phosphorylation were significantly enriched in oncogenic signaling pathways, stromal activation pathways and immune-related signaling pathways (**Figure 5A**). Downregulated proteins were mainly involved in the neuronal system, mitotic cell cycle processes, and so on (**Supplementary Figure S4A**). Further analysis of the combined protein quantitative data showed a significant positive correlation between phosphorylation level and protein expression levels (**Figure 5B** and **Supplementary Table S11**, Pearson r = 0.6527). Analysis of mutation distribution in the TCGA and Wang datasets showed that the PTEN and NF1 mutation rates were greatly increased in both datasets, while the BRAF mutation rate was significantly increased in the Wang dataset (**Figure 3E** and **Supplementary Figure 2C**). Next, we explored specific signaling pathways based on somatic mutations and their downstream alterations (**Figure 5C**). As shown in the heatmap, with increasing GTMEIscore, the expression levels of the tumor suppressor proteins PTEN and NF1 showed a decreasing trend, and we also observed that the phosphorylation levels of the downstream signaling pathway proteins gradually increased (**Figure 5D**). In addition, the tumor mutation distribution analysis showed that the EGFR mutation rate was slightly downregulated in the high GTMEIscore group, and the pathway enrichment analysis showed that proteins with upregulated levels of phosphorylation were significantly enriched in the EGFR tyrosine kinase inhibitor resistance pathway (**Figure 5A**). The gene and protein expression levels of EGFR were also significantly downregulated in the high GTMEIscore group (**Supplementary Table S9**). We next analyzed the relationship between EGFR protein and phosphorylation levels and the GTMEIscore, as shown in **Supplementary Figure S4B**. The phosphorylation levels of EGFR and its downstream proteins were downregulated in the high GTMEIscore group, suggesting that EGFR activation may inhibit the infiltration of immune cells into tumor tissue and that GBM patients with a high GTMEIscore are insensitive to EGFR inhibitors. These results contribute to our understanding of the mechanisms underlying dysregulated protein expression and phosphorylation, pathway dysregulation.

The proteome differential acetylation analysis data showed that compared to the low GTMEIscore group, the high GTMEIscore group showed 459 significantly upregulated acetylation sites in 207 proteins and 605 significantly downregulated acetylation sites in 288 proteins (**Supplementary Table S12**). KEGG and GO biological process (BP) functional annotation analysis revealed that the proteins with different acetylation levels were mainly enriched in metabolism-related signaling pathways, the HIF-1 signaling pathway and immune-related signaling pathways (**Supplementary Figure S4C**). Further correlation analysis with proteomics data showed that the acetylation levels were positively correlated with the protein expression levels (Pearson r = 0.6626), with the levels of acetylation at 317 acetylation sites in 107 proteins being simultaneously upregulated with protein expression levels, the levels of acetylation at 214 acetylation sites in 106 proteins being simultaneously downregulated with protein expression levels (**Figure 5E**, **Supplementary Table S13**). Further KEGG enrichment analysis of these two fractions of altered proteins revealed that they were mainly enriched in metabolism-related and apoptosis-related signaling pathways (**Figure 5F**). Researchers detected more than 30 modified acetylation sites in histones (H1, H2A, H2B, H3.3, and H4) in GBM, and further differential analysis showed that two acetylation sites were significantly upregulated in H4 histones and 25 acetylation sites were significantly downregulated in H2B histones (**Supplementary Table S14**). Further correlation analysis showed that the GTMEIscore was significantly positively correlated with the acetylation level of H4 group proteins and negatively correlated with the acetylation level of H2 group proteins. Notably, some of the H2B acetylation modification levels were positively correlated with the protein expression of CREBBP/EP300 acetyltransferases and some proteins of the BDR family (BRD3/4) and negatively correlated with the GTMEIscore at both the RNA and protein levels; on the other hand, these modification levels were negatively correlated with the expression of the deacetylases HDAC10/11, the expression of which was positively correlated with the GTMEIscore at both the RNA and protein levels (**Figure 5G**, **Supplementary Table S15**). Further survival analysis based on the protein expression levels of these proteins revealed that CREBBP and BRD3 were protective prognostic genes in GBM, while the deacetylase HDAC10 was a prognostic risk factor (**Supplementary 4D**). These data suggest that increased levels of H2B acetylation modification may depend on the activities of CREBBP, BRD3 and HDAC10, which regulate some protective genes to inhibit the infiltration of immune cells.

### Correlation Between the GTMEIscore and Metabolomic and Lipidomic Characteristics

The results of the above analysis revealed that some of the genes and proteins associated with the GTMEIscore might be involved in metabolic pathways (**Figures 4G**, **5A, F**). We thus performed a correlation analysis between the GTMEIscore and tumor metabolite abundance and identified four metabolites that were positively correlated and three metabolites that were negatively correlated with the GTMEIscore (**Figure 5H** and **Supplementary Table S16**, Pearson |r| > 0.3, P value <0.05). Further survival analysis showed that leucine and DL-2-aminoadipic acid (spectral match) were adverse prognostic factors for GBM patients (**Supplementary Figure 5A**), suggesting that they may play an essential role in the TIME.

Next, we analyzed the correlation of the abundances of 582 lipids with the GTMEI score in 75 GBM tumor tissues and found a large number of lipids that were correlated with the GTMEI score (**Supplementary Table S17**, Pearson |r| > 0.3, P value <0.05). As shown in **Figure 5I**, the triacylglycerol (TG) content showed a significant positive correlation with the GTMEIscore and a significant negative correlation with the content of most phosphatidylcholines (PCs) and sphingomyelin (SM).

To further explore the molecules mediating these metabolic changes, we performed a correlation analysis of the GTMEIscore with 29 metabolic regulatory genes previously reported to be associated with GBM prognosis (30) and further performed a survival analysis of these genes based on their protein expression levels. As shown in **Supplementary Figures S5B, C**, ALDH3A1, PSME1 and RUFY1 were adverse prognostic factors that had a significant positive correlation with the GTMEIscore. In contrast, CHD9, PON1 and PON2 were protective prognostic factors. Our results provide valuable insights into the lipid metabolic characteristics of different immune microenvironment patterns in GBM, and reveal possible metabolite targets, regulating the immune microenvironment.

### Correlation of the GTMEIscore With the Efficacy of Immunotherapy and Drug Sensitivity in GBM

**Figure 6:**
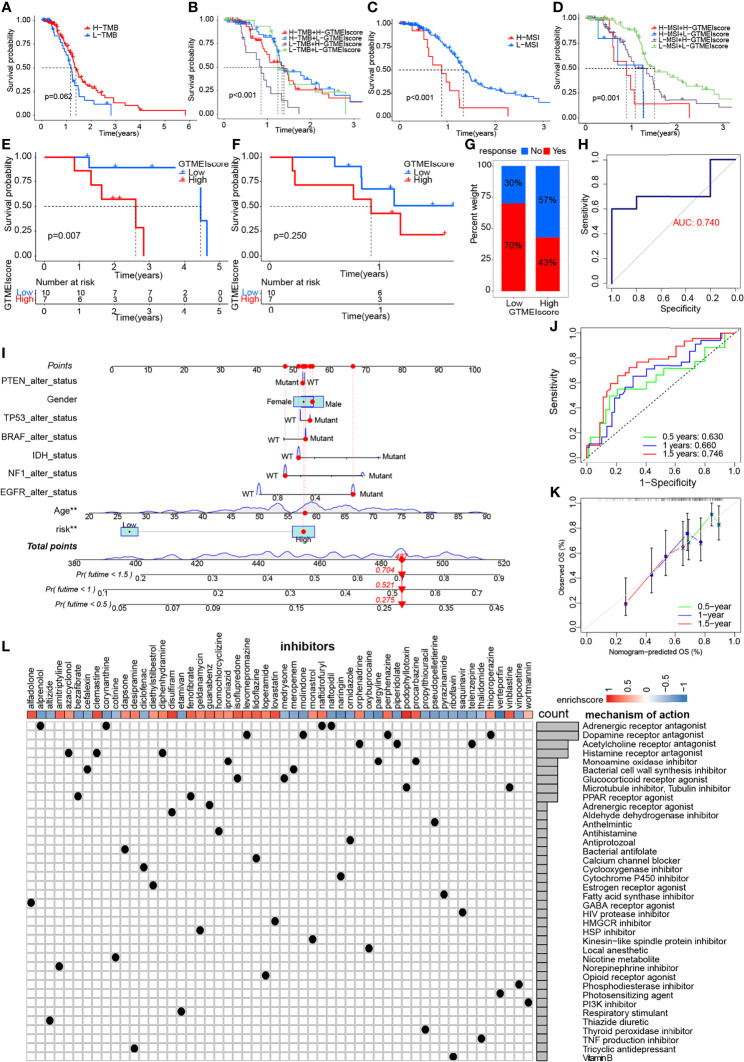
Correlation of the GTMEIscore with the efficacy of immunotherapy and drug sensitivity in GBM. Kaplan-Meier curves for the OS of TCGA GBM patients with **(A)** TMB (log-rank test P = 0.062); **(B)** stratified by GTMEIscore and TMB (log-rank test P < 0.001); **(C)** MSI (log-rank test P < 0.001); and **(D)** GTMEIscore and MSI (log-rank test P = 0.001). Kaplan-Meier curves for **(E)** the OS of GBM patients (log-rank test P = 0.007) and **(F)** survival duration after anti-PD1 treatment (log-rank test P = 0.250 in the PD1 dataset). **(G)** Proportions of patients who responded to anti-PD1 immunotherapy in the low and high GTMEIscore groups. **(H)** ROC curve quantifying the predictive value of the GTMEIscore in GBM patients treated with anti-PD1 therapy (AUC, 0.740). **(I)** A personalized scoring nomogram was constructed to predict the OS probability for 9 parameters at 0.5, 1, and 1.5 years, an example of which is shown by the arrows. **(J)** Time-dependent ROC analysis demonstrated that the nomogram exhibited a powerful capacity for survival prediction. **(K)** Calibration curves showing that the predicted 0.5-year (green dashed line), 1-year (blue dashed line) and 1.5-year (red dashed line) OS values were close to the ideal values (45-degree line). (**L)** CMap mode of action (MoA) analysis revealed a total of 38 mechanisms of action for the 54 compounds significantly related to GTMEIscore, sorted by descending number.

We further evaluated its ability to predict patient response to ICB therapy. Improved response to anti-PD-1 therapy has been found to be associated with higher TMB in tumors across multiple cancer types, including GBM (31). Survival analysis showed that patients with high TMB in the TCGA dataset had significantly better survival than those with low TMB (**Figure 6A**), and further combined GTMEIscore analysis showed that patients with a high GTMEIscore and low TMB had a significant survival disadvantage (**Figure 6B**). Touat et al. (32) recently found that mismatch repair (MMR)-deficient gliomas were characterized by poor patient survival and a low rate of response to anti-PD-1 therapy. Our data also showed that patients with low microsatellite instability (MSI) had significantly better survival than those with high MSI (**Figure 6C**), and further combined GTMEIscore analysis showed a significant survival advantage for patients with a low GTMEIscore and low MSI (**Figure 6D**), suggesting that GTMEIscore combined with markers such as TMB and MSI significantly improved the sensitivity and accuracy, and may be a more effective way to screen the immune beneficiary GBM population. In addition, the GTMEIscore showed a significant positive correlation with immunochekpoint expression in GBM patients at both the RNA and protein levels (**Figures 3F, G**, **Figure 4L**). And survival analysis found that patients with a low GTMEIscore showed a significant clinical advantage and significantly prolonged survival (**Figure 6E**, P=0.007). Similarly, survival, as measured from the start of treatment with anti-PD1 therapy, was slightly increased in GBM patients with a low GTMEIscore (**Figure 6F**, P=0.250). Patients with a low GTMEIscore had significantly increased efficacy of ICI treatment compared to those with a high GTMEIscore (**Figure 6G**, response rate to anti-PD1 therapy: 70% vs. 43%). ROC curve analysis demonstrated good predictive capability of the GTMEIscore in predicting the effectiveness of immunotherapy in GBM patients (**Figure 6H**, AUC=0.740).

To quantify the risk assessment of individual GBM patients, we proposed a comprehensive prognostic nom model using a combination of GTMEIscore combined with other clinicopathological characteristics, an example of which is shown by the arrow (**Figure 6I**). Calibration curves and time-dependent ROC analysis of 0.5, 1 and 1.5-year OS prediction demonstrated the nomogram exhibited much more powerful capacity of survival prediction (**Figures 6J, K**).

Finally, we used the CMap database to predict potential drugs for patients with high GTMEIscore. CMap mode of action (MoA) analysis revealed a total of 38 mechanisms of action for the 54 compounds with significant enrichment (**Figure 6L** and **Supplementary Table S18**). These results provide potential drugs that can be used for patients with a high GTMEIscore.

### Overview of the GTMEIscore Across Human 32 Cancers Types

**Figure 7:**
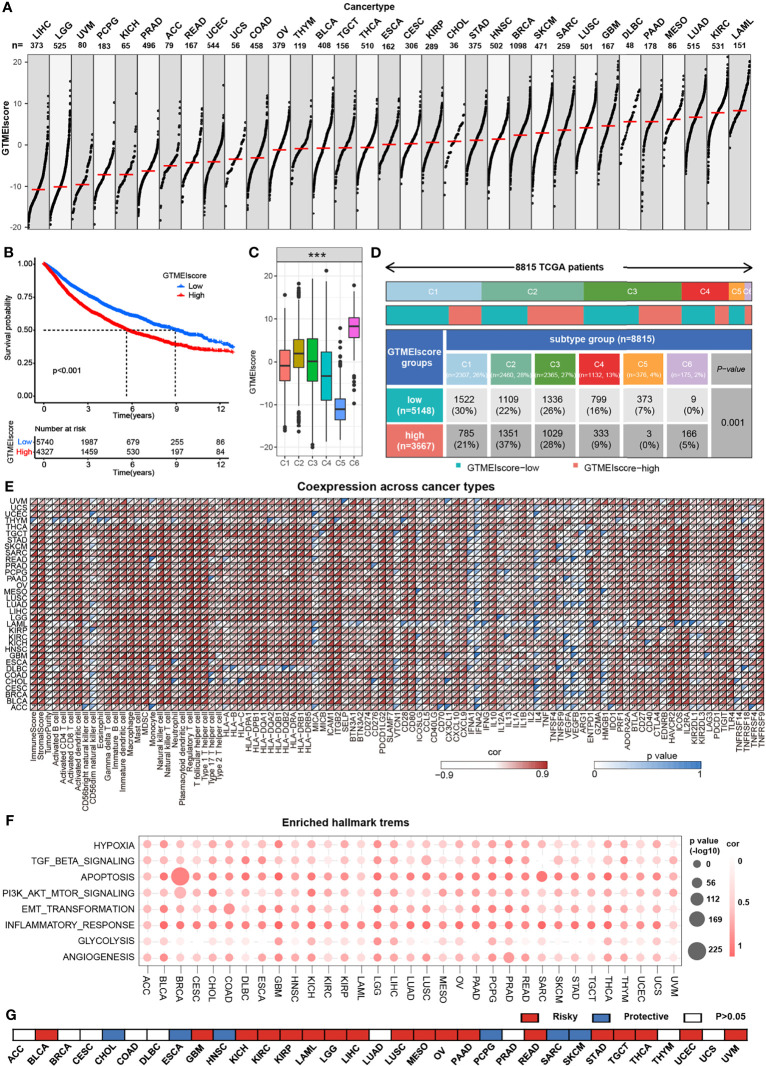
Overview of the GTMEIscore across 32 human cancer types. **(A)** The GTMEIscore for all samples grouped by cancer type, ranked from lowest to highest. **(B)** Kaplan-Meier curves for the OS of 10067 patients with a high GTMEIscore (n = 4327) and a low GTMEIscore (n = 5740), and the log-rank test showed P < 0.001. **(C)** Differences in the GTMEIscore between six different immune molecular subtypes. The Kruskal-Wallis test was used to determine the significance of differences between the six immune molecular subtypes. **(D)** Heatmap and table of the distribution of three immune molecular subtypes between the high and low GTMEIscore groups, chi-square test showed P=0.001. **(E)** Spearman analysis of the correlation of the GTMEIscore with immunomodulators, 28 immune cell types, immune and stromal scores, and tumor purity. Colors indicate correlation coefficients, with yellow indicating a negative correlation and red indicating a positive correlation. Asterisks indicate statistically significant P values calculated using Spearman correlation analysis. *P < 0.05; **P < 0.01; ***P < 0.001. **(F)** Bubble plots showing the correlation between the GTMEIscore and classical cancer pathways. The color of the circle represented the correlation coefficient, and the size represent the p value. **(G)** Summary of the correlation between expression of GTMEIscore and 32 cancer type patients survival. Red represents a higher expression of GTMEIscore associated with worse survival, and blue represents an association with better survival. Only p values < 0.05 are shown.

We further assessed the differences in the GTMEIscore across 33 tumors, and as shown in **Figure 7A**, we found that the GTMEIscore was highest in LAML, followed by KIRP, and lowest in LIHC. Pancancer survival analysis showed that overall survival was significantly shorter in the high GTMEIscore group than in the low group (**Figure 7B**). Differences in the GTMEIscore between different immune subtypes were further investigated. Expression was significantly different between the C1 (wound healing), C2 (IFN-γ dominant, inflammatory), C3 (lymphocyte depleted), C4 (lymphocyte depletion), C5 (immunologically quiet) and C6 (TGF-β dominant) subtypes, which are characterized by differences in macrophage or lymphocyte signatures, and was higher in C2 and C6 subtypes, with poorer prognosis (**Figure 7C** and **Supplementary Figure S6A**). Additionally, Chen et al. (33) proposed three immunophenotypes, namely immune-inflamed, immune-excluded and immune-desert, which are more comparable to the subtypes we obtained (**Supplementary Figures S6B, C**). As shown in **Figure 7D**, the proportion of immune-excluded samples was almost equally distributed between the two groups, but there were more immune-inflamed samples and fewer immune-desert samples in the GTMEI-high group than in the GTMEI -low group (p = 0.001, chi-square test). Further Spearman correlation analysis showed that GTMEIscore was positively correlated with infiltrating immune cells, immune/stromal score and immunomodulatory molecules, while negatively correlated with tumor purity in most tumor types (**Figure 7E**). As shown in **Figure 7F**, we also found that GTMEIscore was positively correlated with in most tumors, indicating that the GTMEIscore is associated with enrichment scores of typical cancer hallmarks in a wide range of cancer types. The cancer-specific survival analysis also revealed a significant association between the GTMEIscore and overall survival in multiple cancer types: the GTMEIscore was a risk factor in 17 cancer types, and a protective factor in 7 cancer types (**Figure 7G** and **Supplementary Figure S7**). These results demonstrated the characteristics of the GTMEIscore in a broad range of cancer types and highlight its potential value as a predictor of immune cell infiltration and prognosis.

**Figure 8:**
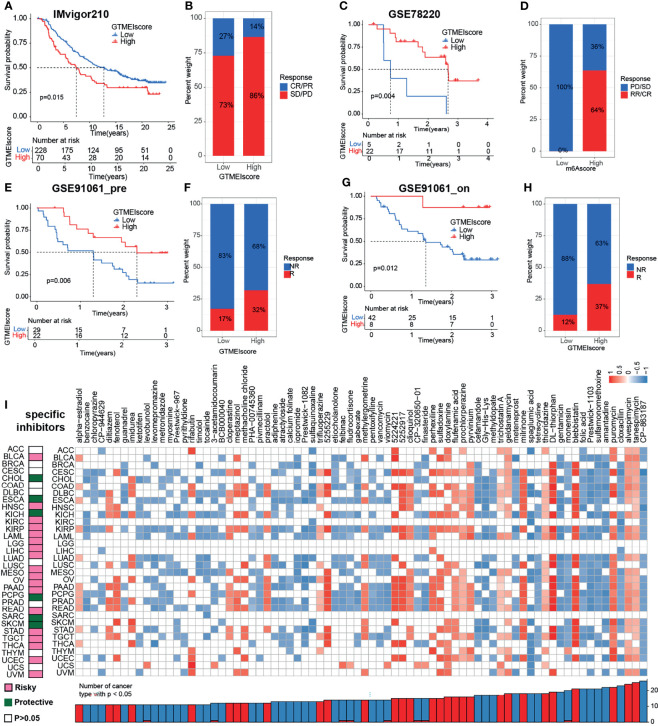
Correlation of the GTMEIscore with the efficacy of immunotherapy and drug sensitivity in other 32 cancer types. **(A)** The Kaplan–Meier survival curves showed that GTMEIscore was a prognostic risk factor in the urothelial cancer patients in IMvigor210 dataset that received anti-PD1 therapy(log-rank test P = 0.015). **(B)** Proportions of urothelial cancer patients who responded to anti-PD1 immunotherapy in the low and high GTMEIscore groups in IMvigor210 dataset. **(C)** The Kaplan–Meier survival curves showed that GTMEIscore was a prognostic protective factor in the melanoma patients in GSE78220 dataset that received anti-PD1 therapy(log-rank test P = 0.004). **(D)** Proportions of melanoma patients who responded to anti-PD1 immunotherapy in the low and high GTMEIscore groups in GSE78220 dataset. **(E)** The Kaplan–Meier survival curves showed that GTMEIscore was a prognostic protective factor in the melanoma patients in GSE91061 dataset that had not received anti-PD1 therapy (log-rank test P = 0.006). **(F)** Proportions of melanoma patients who responded to anti-PD1 immunotherapy in the low and high GTMEIscore groups in GSE91061 dataset that had not received anti-PD1 therap. **(G)** The Kaplan–Meier survival curves showed that GTMEIscore was a prognostic protective factor in the melanoma patients in GSE91061 dataset that received anti-PD1 therapy (log-rank test P = 0.012). **(H)** Proportions of melanoma patients who responded to anti-PD1 immunotherapy in the low and high GTMEIscore groups in GSE91061 dataset received anti-PD1 therapy. **(I)** The left heatmap showing the summary of the correlation between expression of GTMEIscore and other 32 cancer type patients survival. Pink represents a higher expression of GTMEIscore associated with worse survival, and green represents an association with better survival. Only p values < 0.05 are shown. The right heatmap showing the enrichment fraction of each compound in CMap for other 32 cancer types (positive in blue, negative in red). Compounds are sorted from right to left by decreasing number of significantly enriched cancer types.

We further investigated the predictive power of GTMEIscore for other cancer types of ICB therapy. Thus, we used urothelial cancer cohorts of patients (IMvigor210) and two cohorts of melanoma patients who received anti-PD1 therapy (22, 23) to perform a complementary evaluation of the ability of GTMEIscore to predict the immunotherapy response. GTMEIscore was a risky factor in Urothelial Carcinoma, while was a protective factor in melanoma (**Figure 7G**). And survival analysis found that urothelial cancer patients with a low GTMEIscore showed a significant clinical advantage and significantly prolonged survival (**Figure 8A**, P=0.015), and had significantly increased efficacy of ICI treatment compared to those with a high GTMEIscore (**Figure 8B**, response rate to anti-PD1 therapy: 27% vs. 14%). However, survival analysis found that melanoma patients with a high GTMEIscore showed a significant clinical advantage and significantly prolonged survival (**Figure 8C, E, G**, P=0.015, 0.006 and 0.012, respectively), and had significantly increased efficacy of ICI treatment compared to those with a low GTMEIscore (**Figure 8D, F, H**, response rate to anti-PD1 therapy: 64% vs. 0%, 32% vs. 17% and 37% vs. 12%, respectively).

To further understand the impact of the GTMEIscore on drug response in 32 cancer types, we obtained the differential genes for each cancer type in GTMEIscore high group by grouping according to the optimal survival cut value (**Supplementary Table S19**), and further predicted the relevant compounds by inputting the most significantly up- and down-regulated 1000 genes to the cmap database. We show only the compounds found to be significantly associated with GTMEIscore in at least ten cancer types (**Figure 8I** and **Supplementary Table S20A**). Tumors with a better prognosis are sensitive to drugs with a high enrichscore and conversely tumors with a poor prognosis are sensitive to drugs with a low enrichscore. Further, we demonstrated the targeting mechanism of these drugs using MOA analysis of cmap (**Supplementary Figure S8A** and **Table S20B**). These results provide potential drugs that can be used for patients with a high GTMEIscore, offering potential therapeutic prospects for improving the prognosis of cancer patients.

## Discussion

Recent advances in tumor immunotherapy have created great enthusiasm and anticipation for the effective treatment of GBM (20, 34). The TME, especially the TIME, plays an important role in clinical survival and response to therapy as a key mediator of tumor progression and treatment outcome (9–11, 35). Analyzing the TIME of GBM patients may provide new insights into the development of immunotherapeutic strategies for GBM. In this study, we integrated transcriptome information from 796 GBM samples, used single sample gene-set enrichment analysis (ssGSEA) to estimate the relative abundances of 25 immune cell types, and provided a comprehensive outlook on the immune landscape within GBM tumors. We revealed three distinct GTMEI patterns with different clinical outcomes and modulated biological pathways. Moreover, we developed a scoring system to quantify the immune infiltration pattern of individual GBM sample, termed GTMEIscore. To understand the intrinsic tumor characteristics and tumor immune infiltration patterns associated with the GTMEIscore, we comprehensively analyzed the genomic characteristics, molecular subtypes and clinicopathological features as well as proteomic, phosphoproteomic, acetylomic, lipidomic and metabolomic properties associated with the GTMEIscore, revealing lots of novel dysregulated pathways and precise targets in GBM. Moreover, GTMEIscore accurately quantified the immune status of many other cancer types. Clinically, GTMEIscore was found to have significant potential therapeutic value for chemotherapy/radiotherapy, immune checkpoint inhibitor (ICI) therapy and targeted therapy. Our systematic identification and characterization of molecular subtypes of the immune microenvironment of GBM revealed many novel dysregulated signaling pathways and precise targets in GBM and provides a theoretical basis for identifying more effective predictive biomarkers and developing more effective and targeted clinical treatment strategies for GBM.

This paper highlights the important role of TME, particularly macrophages, in shaping the MES-like cellular state. Several immune microenvironment studies for other tumors display better prognosis for immune inflamed subtype which more effective immune infiltration in tumor stroma (12, 17). However, the result of immune infiltration in GBM, the GTMEI cluster C which had better prognosis did not show the increased immune infiltration and the activated immune state, which may be due to the special intracranial microenvironment. Recently, using scRNA-seq, malignant cells in GBM were classified into four potentially plastic cell states: neural progenitor cell-like (NPC-like), oligodendrocyte progenitor cell-like (OPC-like), astrocyte-like (AC-like), and mesenchymal-like (MES-like) (35). Hara et al. (36) further showed striking similarities between the MES-like state and the TCGA-MES subtype; both were rich in macrophages, and the GBM MES-like state was also associated with increased abundance and cytotoxicity of T cells. Our study showed that the characteristics of the immunoinflammatory subtypes (GTMEI clusterA) identified in our study are also highly similar to those of the previously reported MES subtypes. Whereas Ester Gangoso et al. (10) recently found that a key component of the previously reported “mesenchymal” signature is the transcriptional module acquired in GBM cells after immune attack, the observed transformation of GBM tumor subtypes can be explained by the extent to which the tumor immune microenvironment encroaches on their epigenetic landscape and alters the regulatory network of transcription factors. However, these results suggest that, at least in glioblastoma, the MES-like status of macrophages and cancer cells may also represent a therapeutic opportunity, as they are associated with high levels T cells that tend to be in a cytotoxic state, which may affect the response to immunotherapy. Thus, induction of MES-like states by safe and effective means and in combination with immunotherapeutic approaches may provide a new therapeutic option.

Genomic alterations and alterations in downstream oncogenic signaling pathways in tumors have been shown to affect antitumor immunity and TME activity (18, 20, 26). As such, we investigated the link between tumor mutations and the GTMEIscore and found that compared to the low GTMEIscore group, the GTMEIscore group had significantly higher PTEN and NF1 mutation rates (**Figure 3E** and **Supplementary Figure 2C**), and PTEN and NF1 mutations have been shown to cause increased infiltration of TAMs into tumor tissue (26, 27, 35). Single-cell sequencing studies have shown that TAMs are the most abundant component of the GBM immune microenvironment, originating from two independent sources (BMDM TAMs and MG TAMs) (9, 11), and they respond differently at different stages of tumor progression and perform different functions (37). These differences may be partly explained by the fact that the two cell populations are derived from different progenitor cells, which are selectively distributed in different locations, and employ different TFs for gene regulation (37). Numerous studies have demonstrated that the macrophage population that exerts immunosuppressive and proangiogenic effects is generally of bone marrow origin (9, 10, 26, 27), and our study found that the GTMEIscore was positively correlated with TAM BMDMs and negatively correlated with TAM MGs (**Figure 2J**). We also identified and validated a significant positive correlation between the GTMEIscore and myeloid-derived macrophage-restricted chemokines and genes encoding ECM and matricellular proteins (**Figures 2F, G**, **4L**). Our results showed that the GTMEIscore predicted GBM heterogeneity as well as the functional status of macrophages. Therefore, considering the significant differences in biological functions, TAM components and T cell abundance among GBM infiltrating immune cell subtypes, this study may provide more ideas for the future development of subtype-specific combination immunotherapy strategies.

Proteomic analysis revealed a large number of differentially expressed proteins in GBM tumors with different immune infiltration patterns, and some tumors showed significant downregulation of cell cycle- and DNA repair-related proteins and upregulation of apoptosis-, EMT-, metabolism- and immune response-related proteins (**Figure 4**), in line with the transcriptomic analysis results (**Figure 3**). Furthermore, phosphoproteomics analysis also identified a large number of dysregulated protein phosphorylation sites in GBM tumors with different immune infiltration patterns, revealing a number of proteins associated with apoptosis, ECM, metabolism, and the immune response and further providing candidates for targeted therapy of GBM (**Figures 5A, B**). In addition, somatic mutation analysis of the high GTMEIscore group revealed significantly increased rates of PTEN, NF1 and BRAF mutation, and subsequent proteomics and phosphoproteomics analysis revealed dysregulation of downstream signaling pathway proteins and phosphorylation sites (**Figures 5C, D**). We also characterized the acetylation patterns of tumors with different immune infiltration patterns, revealing a large number of proteins with dysregulated acetylation, which were mainly involved in metabolic pathways (**Figures 5E, F**). In addition, analysis of histone modifications revealed significant downregulation of multiple acetylation site modification levels in H2B histones in the high GTMEIscore group, which may be dependent on CREBBP/EP300/BRD3/BRD4 activity (**Figure 5G**). For the first time, we employed a multilevel and multiplatform strategy to construct a multidimensional molecular map of tumors with different immune infiltration patterns. The results will help comprehensively reveal the molecular mechanisms of GBM development and immune microenvironment dysregulation, and provide an important scientific basis for improving the clinical treatment and prognosis of GBM.

In addition, we also demonstrated the immunomodulatory landscape in other 32 cancer types with a TCGA dataset and found significant correlations of the GTMEIscore with the immune status and biological functions of most tumors (**Figure 7**). We demonstrated that the GTMEIscore can be used not only as an independent prognostic biomarker for predicting survival in GBM, BLCA and SKCM patients but also for predicting the response to anti-PD1 antibody immunotherapy in these cancers. Drug sensitivity is a constant factor at the core of individualized cancer chemotherapy, we also predicted potential drugs that can be used for patients with a high GTMEIscore for 33 cancer types (**Figure 8**). TGF-beta is an important factor contributing to PD-L1/PD-1 antibody resistance by limiting T cell infiltration in the TME. Therefore, blocking TGF-beta significantly enhanced the efficacy of anti-PD-1/PD-L1. Recently, bispecific antibodies targeting TGF-Beta and PD-L1 exhibited superior antitumor activity (38–40), indicating that the combination of these drugs with ICIs, as well as antibodies targeting TGF-Beta may have better therapeutic effects for patients.

In conclusion, our systematic identification and characterization of molecular subtypes of immune microenvironments in GBM revealed many novel dysregulated signaling pathways and precise targets in GBM. Based on this multiomics data study, we found that the GTMEIscore is a reliable prognostic biomarker that can robustly predict the effect of ICIs and combination therapy with chemotherapy/radiotherapy. These findings might provide a theoretical basis for identifying more effective GBM predictive biomarkers and developing more effective and targeted clinical treatment strategies, ultimately guiding GBM clinical treatment and achieving precision medicine.

## Data Availability Statement

All data used in this work can be acquired from the Gene-Expression Omnibus (GEO; https://www.ncbi.nlm.nih.gov/geo/) under the accession numbers GSE78220 and GSE91061, CGGA (http://www.cgga.org.cn/), GlioVis database (http://gliovis.bioinfo.cnio.es/), the TCGA GDC portal (https://portal.gdc.cancer.gov/repository), https://www.ncbi.nlm.nih.gov/sra/ under the accession number PRJNA482620, and supplemental data of the Corresponding article.

## Author Contributions

GL and HX supervised the project. RRZ and WZP designed the research and executed all the results. BYL, SLZ, SJZ, YHQ, JWQ, ZJG, YF, QDG, WQ, SBW and QTW helped to revise the manuscript. PZ, XG, and LD provided administrative and technical support. All authors read and approved the final manuscript.

## Funding

This work was supported by grants from the National Natural Science Foundation of China (Nos. 81874083; 82072776; 82072775; 81702468; 81802966; 81902540; 81874082; 81472353), Natural Science Foundation of Shandong Province of China (Nos. ZR2019BH057; ZR2020QH174; ZR2021LSW025), the Jinan Science and Technology Bureau of Shandong Province (2021GXRC029), Key Clinical Research Project of Clinical Research Center of Shandong University (2020SDUCRCA011) and Taishan Pandeng Scholar Program of Shandong Province (No. tspd20210322).

## Conflict of Interest

The authors declare that the research was conducted in the absence of any commercial or financial relationships that could be construed as a potential conflict of interest.

## Publisher’s Note

All claims expressed in this article are solely those of the authors and do not necessarily represent those of their affiliated organizations, or those of the publisher, the editors and the reviewers. Any product that may be evaluated in this article, or claim that may be made by its manufacturer, is not guaranteed or endorsed by the publisher.
