# Supplementary material for: Comprehensive Analysis of the Tumor Immune Microenvironment Landscape in Glioblastoma Reveals Tumor Heterogeneity and Implications for Prognosis and Immunotherapy
Source: Front Immunol. 2022 Mar 2;13:820673. doi: 10.3389/fimmu.2022.820673 (PMC8924366; doi:10.3389/fimmu.2022.820673)
Supplement: Supplementary file 1 [file Table_1.docx]

**Supplementary methods**

# Collection and preprocessing of publicly available pan-cancer expression datasets

The RNA-seq data (RPKM format) and corresponding clinical information for patients with 32 other types of cancer (ACC: adrenocortical carcinoma; BLCA: bladder urothelial carcinoma; BRCA: breast invasive carcinoma; CESC: cervical squamous cell carcinoma; CHOL: cholangiocarcinoma; COAD: colon adenocarcinoma; DLBC: lymphoid neoplasm diffuse large B cell lymphoma; ESCA: esophageal carcinoma; LGG: brain lower grade glioma; HNSC: head and neck squamous cell carcinoma; KICH: kidney chromophobe; KIRC: kidney renal clear cell carcinoma; KIRP: kidney renal papillary cell carcinoma; LAML: acute myeloid leukemia; LIHC: liver hepatocellular carcinoma; LUAD: lung adenocarcinoma; LUSC: lung squamous cell carcinoma; MESO: mesothelioma; OV: ovarian serous cystadenocarcinoma; PAAD: pancreatic adenocarcinoma; PCPG: pheochromocytoma and paraganglioma; PRAD: prostate adenocarcinoma; READ: rectum adenocarcinoma; SARC: sarcoma; SKCM: skin cutaneous melanoma; STAD: stomach adenocarcinoma; TGCT: testicular germ cell tumor; THCA: thyroid carcinoma; THYM: Thymoma; UCEC: uterine corpus endometrial carcinoma; UCS: uterine carcinosarcoma; and UVM: uveal melanoma) were obtained from TCGA. All expression data were then transformed into TPM format.

**Consensus clustering for 25 TME immune cells**

Unsupervised clustering was used to identify different immune microenvironment patterns based on the enrichment abundances of 25 infiltrating immune cells and to classify patients into different clusters for further analysis. We applied the “ConsensuClusterPlus” package^[1]^ to perform the above steps and repeated the analysis 1000 times to ensure the stability of the classification.

**Gene set variation analysis (GSVA) and biological function annotation**

To investigate the differences in the biological functions of the different immune microenvironment patterns, we performed functional enrichment analysis of each sample using the "GSVA" R package^[2]^. We performed gene annotation enrichment analysis for the indicated genes using the “clusterProfiler” R package^[3]^ or Metascape database (https://metascape.org/gp/index.html). We also identified differentially activated biological pathways between the high and low groups by performing gene set enrichment analysis (GSEA) of the overall expression data of all transcripts or proteins. The KEGG and hallmark gene sets were downloaded from MSigDB, and the other related biological process gene sets were obtained from a study of Mariathasan S et al.^[4]^.

**Differentially expressed genes (DEGs) associated with GTMEI patterns**

To identify genes associated with different immune microenvironments, we used the empirical Bayesian approach of the “limma” R package^[5]^ to determine the DEGs between three different GTMEI patterns. The criteria for determining DEGs were set as adjusted P value < 0.0001 and fold change (FC) >2.

**Dimension reduction and generation of the GTMEIscore system**

To determine the immune infiltration pattern of individual tumors, we constructed a scoring system to assess the immune microenvironment pattern of individual GBM patients, termed GTMEIscore. The construction was accomplished as follows. First, DEGs identified in the different GTMEI patterns were normalized in the combined GBM dataset samples to extract overlapping DEGs, and then these genes were subjected to prognostic analysis using univariate Cox regression analysis. Then, genes with significant prognostic value (P<0.001) were extracted for further analysis to reduce noise and redundant genes^[6]^, and consensus clustering analysis of the genes with significant prognostic value was utilized to define the number of GTMEI gene clusters as well as their stability. Next, we extracted genes with significant prognostic value and defined them as the GTMEI gene signature for further analysis. We performed principal component analysis (PCA) to construct the GTMEI-related gene signature. PCA1 and PCA2 were extracted to act as the gene signature score. Finally, we applied a method similar to GGI^[7]^ to define the GTMEIscore of each patient: GTMEIscore= Σ(PC1i+PC2i), where i is the expression of a GTMEI-related gene.

# Tumor mutation distribution analysis

The corresponding mutation data of patients in the TCGA-GBM cohort were downloaded from the TCGA data portal (<https://www.cancer.gov/tcga/>). To determine the tumor mutation burden (TMB) of GBM, we counted the total number of nonsynonymous mutations. The corresponding mutation data of patients in the Wang cohort were obtained from the supplementary data of the study. The waterfall function of the “maftools” package was used to show the difference in mutation distribution between patients in the high and low GTMEIscore groups.

# Estimation of stromal cells aboundence

The proportion of stromal cells was estimated using the Microenvironment Cell Populations-counter (MCP-counter) method, which robustly quantifies the absolute abundance of 2 stromal cell populations (fibroblasts and endothelial cells) in heterogeneous tissues from transcriptomic data.

# Calculation of the MES score

Wang L et.^[8]^ used TCGA RNA-seq data to categorize GBM into three subtypes: PN, MES, and CL. We obtained the gene set for the MES signature from the article supplementary data and quantified the MES score for each GBM sample using the ssGSEA algorithm.

# Integrated transcriptome and proteome analysis

The proteomic, phosphoproteomic, acetylomic, lipidomic and metabolomic data in the Wang dataset were acquired from the supplemental data of the paper.

Transcriptome and proteomic data in the Wang dataset were acquired from the supplemental data of the paper. The differentially expressed mRNAs and proteins between the high and low GTMEIscore groups were screened by using the “limma” package in R language. For transcriptome data, a P value<0.05 and |FC|> 2 were considered the cutoff values for determining DEGs. For proteomic data, a P value<0.05 and |FC|>1.2 were considered the cutoff values for determining differentially expressed proteins. A nine-quadrant diagram showing genes with transcriptional and translational expression differences was constructed.

# Integrated proteome and phosphoproteomics/acetylomics analysis

The differential phosphosite/acetylomics abundances between the high and low GTMEIscore groups were also analyzed by using the “limma” package in R language. P value<0.05 and |FC|>1.2 were used as the cutoff values for determining differential phosphosite/acetylomics values, and the proteins with significantly upregulated or downregulated phosphosite/acetylomic values were considered as differential phosphoproteins. The overall trends of the relationships between protein phosphorylation/acetylation levels and their corresponding protein expression and the distribution of differential phosphorylation/acetylation sites and their corresponding protein expression differences were visualized by two histologies distributed in two dimensions (i.e., the horizontal X-axis and vertical Y-axis).

# Construction of integrated prognostic models

Using R package ‘rms’, a scoring nomogram was generated with detailed parameters including age, gender, PTEN/TP53/BRAF/IDH/NF1/EGFR status, and GTMEIscore. 0.5, 1-year, and 1.5-year calibration curves were plotted and compared to actual OS. The effectiveness of the model's predictions was further assessed by ROS curve analysis

# Compounds Targeting with GTMEIscore

To correlate drugs with GTMEIscore, we used the Connectivity Map (also known as cmap) database, a public online tool (https://portals.broadinstitute.org/cmap/) that allows users to predict whether a compound can activate or repress a gene expression-based signature^[9]^. We selected the top 1000 genes of differentially expressed up- or down-regulated genes (500 up-regulated and 500 down-regulated) as representative differential genes to input into the database species to compare predicted drugs.

To further investigate about mechanism of actions (MoA) and drug-target we performed specific analysis within Connectivity Map tools (https://clue.io/)^[10]^.

# Correlation analysis of the GTMEIscore and drug sensitivity

Drug sensitivity data (Zscore values) and gene expression data were downloaded from the CellMiner database (https://discover.nci.nih.gov/cellminer/), and the drugs were screened. Any data that were not FDA approved and were not from clinical trials were excluded. In addition, we downloaded drug sensitivity data (AUC values) of the drugs in cancer cells and annotation data of the targeting pathways of the related drugs from the GDSC database (https://www.cancerrxgene.org/) and RNA-seq data of the corresponding cell lines from the CCLE database (https://sites.broadinstitute.org/ccle/). We then performed Pearson correlation analysis to calculate the correlation between drug sensitivity and the GTMEIscore, and a P value < 0.05 was considered significant.

# References

1. Wilkerson, M. and D. Hayes, *ConsensusClusterPlus: a class discovery tool with confidence assessments and item tracking.* Bioinformatics (Oxford, England), 2010. **26**(12): p. 1572-3.

2. Hänzelmann, S., R. Castelo, and J. Guinney, *GSVA: gene set variation analysis for microarray and RNA-seq data.* BMC bioinformatics, 2013. **14**: p. 7.

3. Yu, G., et al., *clusterProfiler: an R package for comparing biological themes among gene clusters.* OMICS, 2012. **16**(5): p. 284-7.

4. Mariathasan, S., et al., *TGFβ attenuates tumour response to PD-L1 blockade by contributing to exclusion of T cells.* Nature, 2018. **554**(7693): p. 544-548.

5. Ritchie, M., et al., *limma powers differential expression analyses for RNA-sequencing and microarray studies.* Nucleic acids research, 2015. **43**(7): p. e47.

6. Speiser, J., et al., *A Comparison of Random Forest Variable Selection Methods for Classification Prediction Modeling.* Expert systems with applications, 2019. **134**: p. 93-101.

7. Sotiriou, C., et al., *Gene expression profiling in breast cancer: understanding the molecular basis of histologic grade to improve prognosis.* Journal of the National Cancer Institute, 2006. **98**(4): p. 262-72.

8. Wang, Q., et al., *Tumor Evolution of Glioma-Intrinsic Gene Expression Subtypes Associates with Immunological Changes in the Microenvironment.* Cancer cell, 2017. **32**(1): p. 42-56.e6.

9. Lamb, J., et al., *The Connectivity Map: using gene-expression signatures to connect small molecules, genes, and disease.* Science (New York, N.Y.), 2006. **313**(5795): p. 1929-35.

10. Subramanian, A., et al., *A Next Generation Connectivity Map: L1000 Platform and the First 1,000,000 Profiles.* Cell, 2017. **171**(6): p. 1437-1452.e17.
