# Supplementary material for: Comprehensive Analysis of the Tumor Immune Microenvironment Landscape in Glioblastoma Reveals Tumor Heterogeneity and Implications for Prognosis and Immunotherapy
Source: Front Immunol. 2022 Mar 2;13:820673. doi: 10.3389/fimmu.2022.820673 (PMC8924366; doi:10.3389/fimmu.2022.820673)
Supplement: Supplementary file 2 [file Table_2.docx]

**Supplementary Figures**


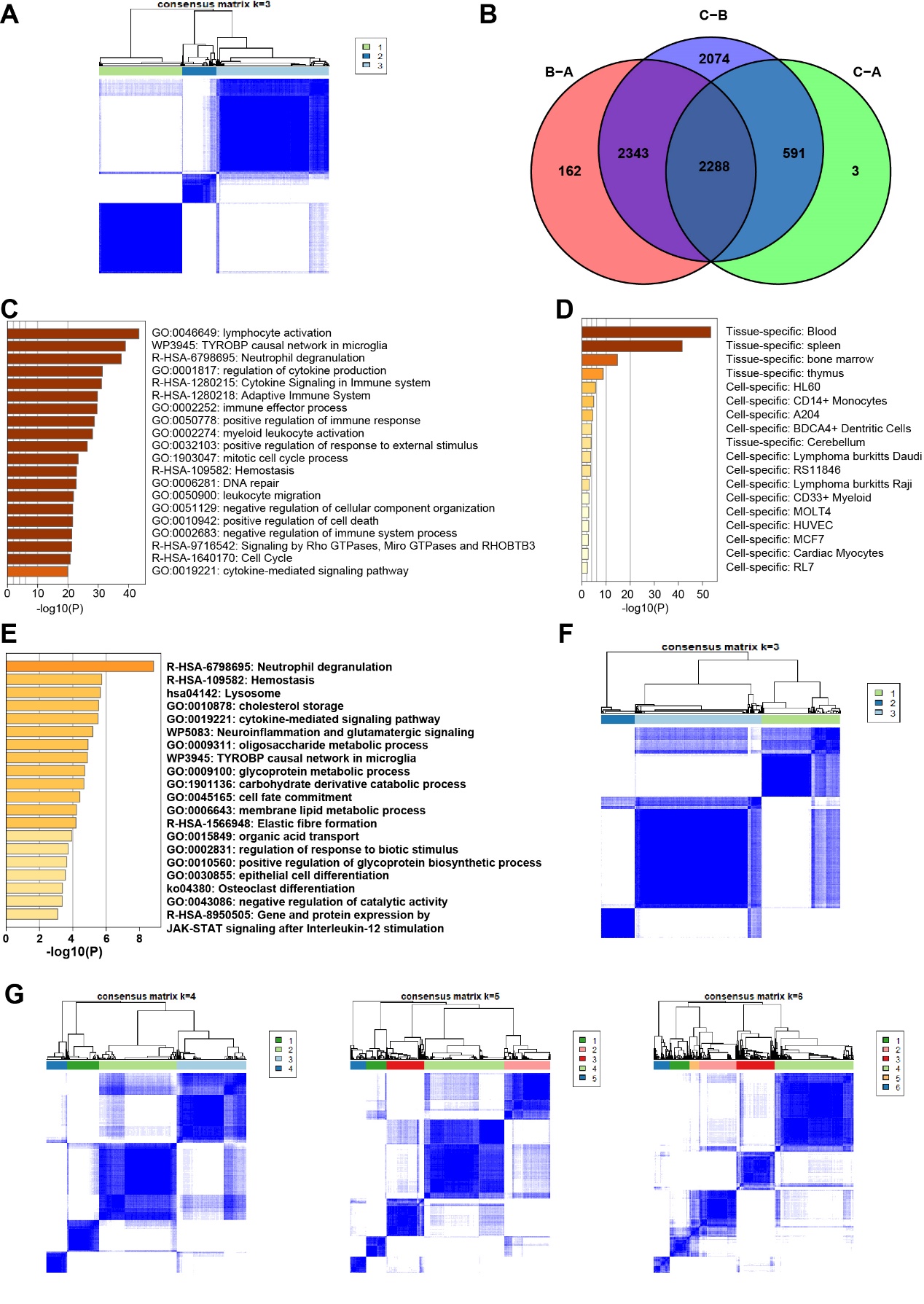


**Supplementary Figure 1. Generation of the GTMEI gene signature and functional annotation.** **A** Consensus matrix of 796 GBM samples with K=3, displaying the stability of the GTMEI clusters using 1000 iterations of hierarchical clustering. **B** Venn diagram showing the number of DEGs among the three GTMEI clusters. (**C**) Bar graph of enriched terms colored by P value and (**D**) summary of the PaGenBase enrichment analysis of DEGs among the three GTMEI clusters. E Bar graph of the enriched terms across the 135 most representative DEGs among the three GTMEI clusters colored by P value. **F** Consensus matrixes of the 796 GBM samples with K=3, displaying the stability of the GTMEI gene clusters using 1000 iterations of hierarchical clustering. **G** Consensus matrixes of the 796 GBM samples with K=4, 5 and 6.


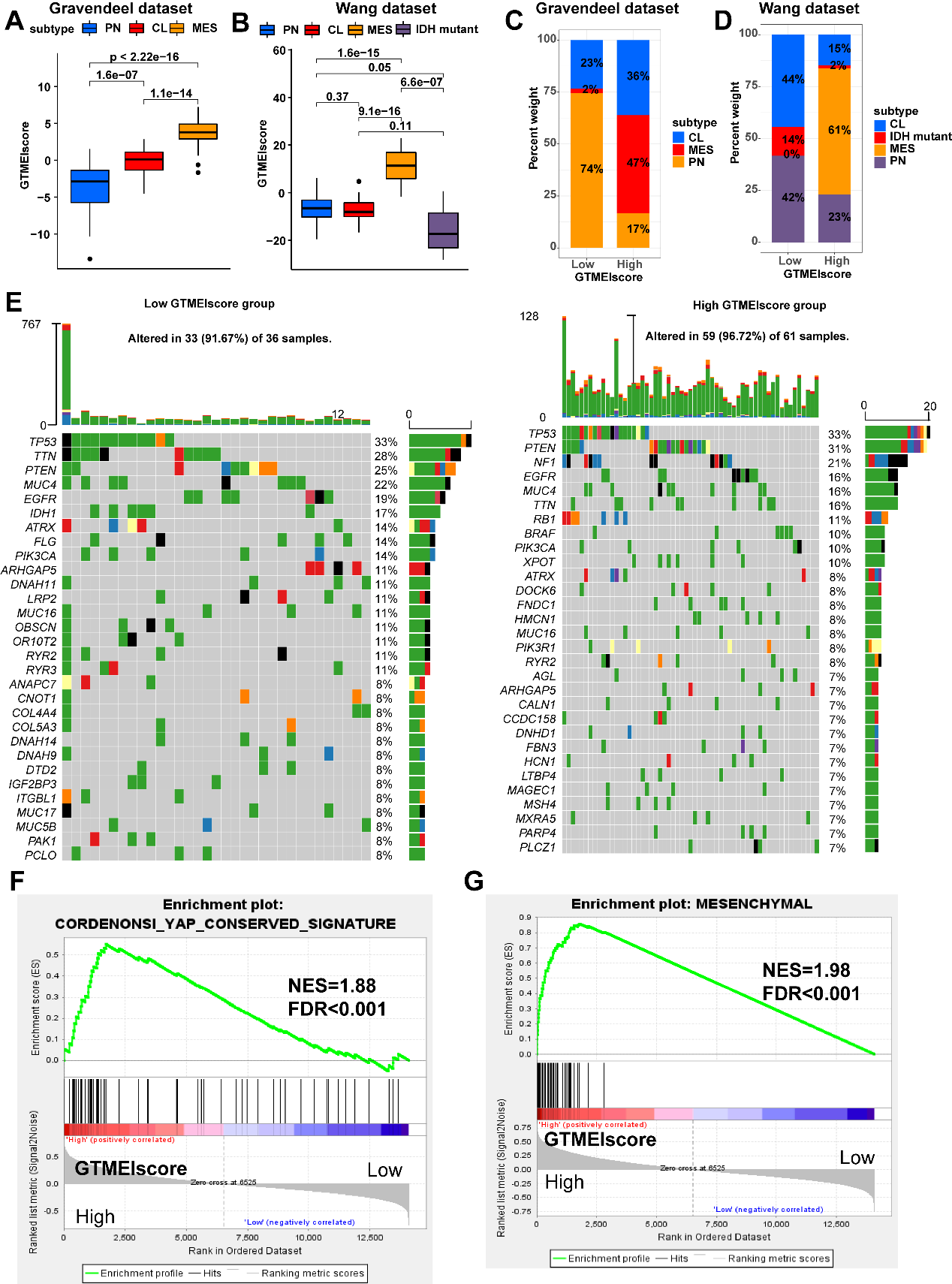


**Supplementary Figure 2 Molecular subtypes tumor somatic mutations associated with the GTMEIscore.** Differences in the GTMEIscore between different GBM molecular subtypes in the (**A**) Gravendeel dataset and (**B**) Wang dataset. The Kruskal-Wallis test was used to determine the significance of differences between the three GBM molecular subtypes. **E** Waterfall plot of the tumor somatic mutation landscape in the low GTMEIscore (left) and high GTMEIscore (right) groups in the Wang dataset. Each bar represents the mutation information for an individual patient. The top bar plot shows TMB, and the numbers on the right indicate the mutation frequency of each gene. The bar plot on the right shows the proportion of each mutation type. GSEA with the (**F**) CORDENONSI_YAP_CONSERVED_SIGNATURE signature and (**G**) mesenchymal signature showed that GBM samples with a high GTMEIscore were enriched in the MES-subtype group compared to GBM samples with a low GTMEIscore group. NES, normalized enrichment score; FDR, false discovery rate.


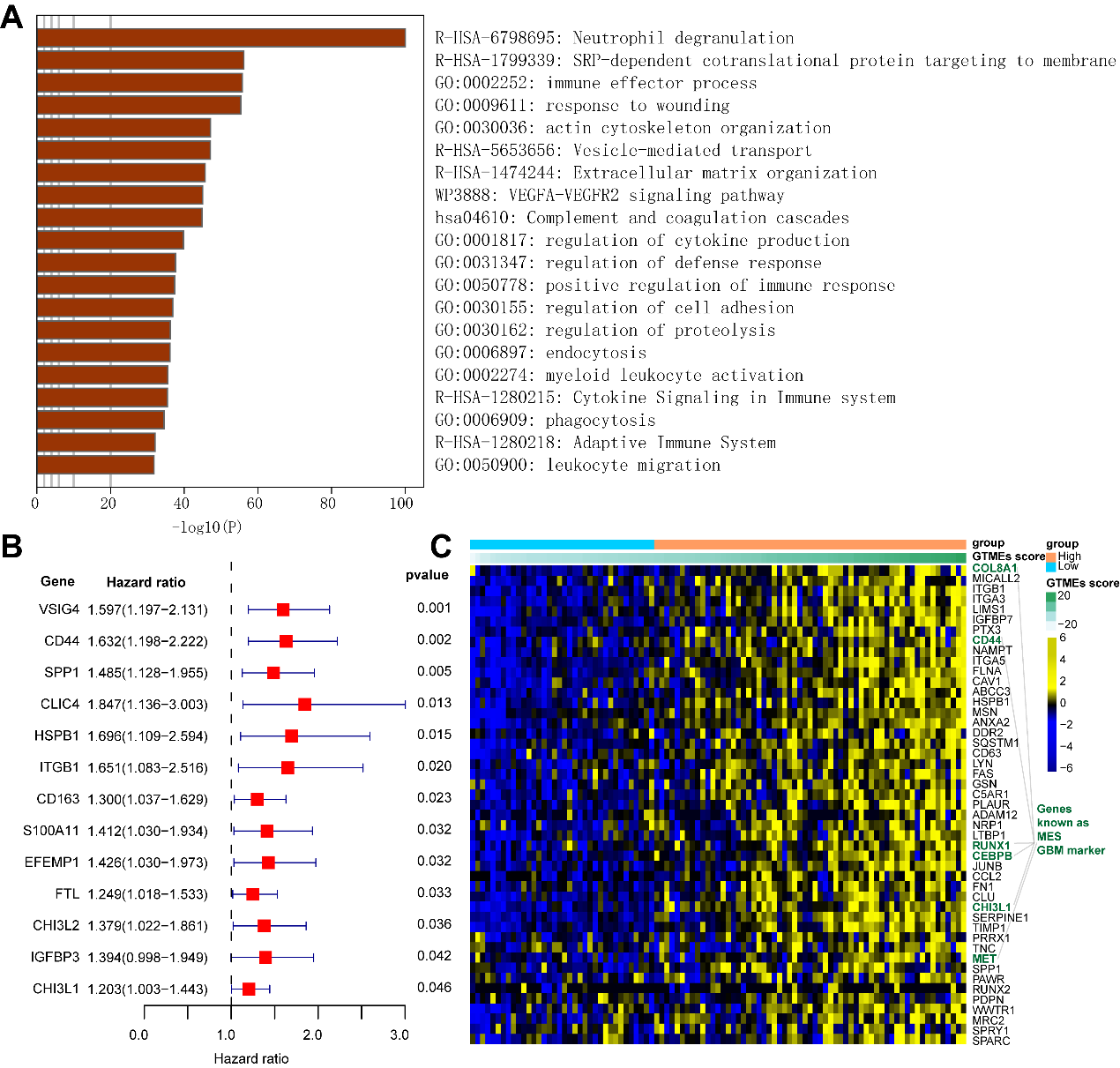


**Supplementary Figure 3 Correlation between the GTMEIscore and proteomic characteristics. A** Bar graph of enriched terms across proteins with a significant positive association with the GTMEIscore colored by P value. **B** Forest plot showing proteins with prognostic significance. An HR > 1.0 indicates that the protein is a prognostic risk biomarker. **C** Heatmap showing the protein expression of genes upregulated in the nmf2-subtype group in the Wang dataset.


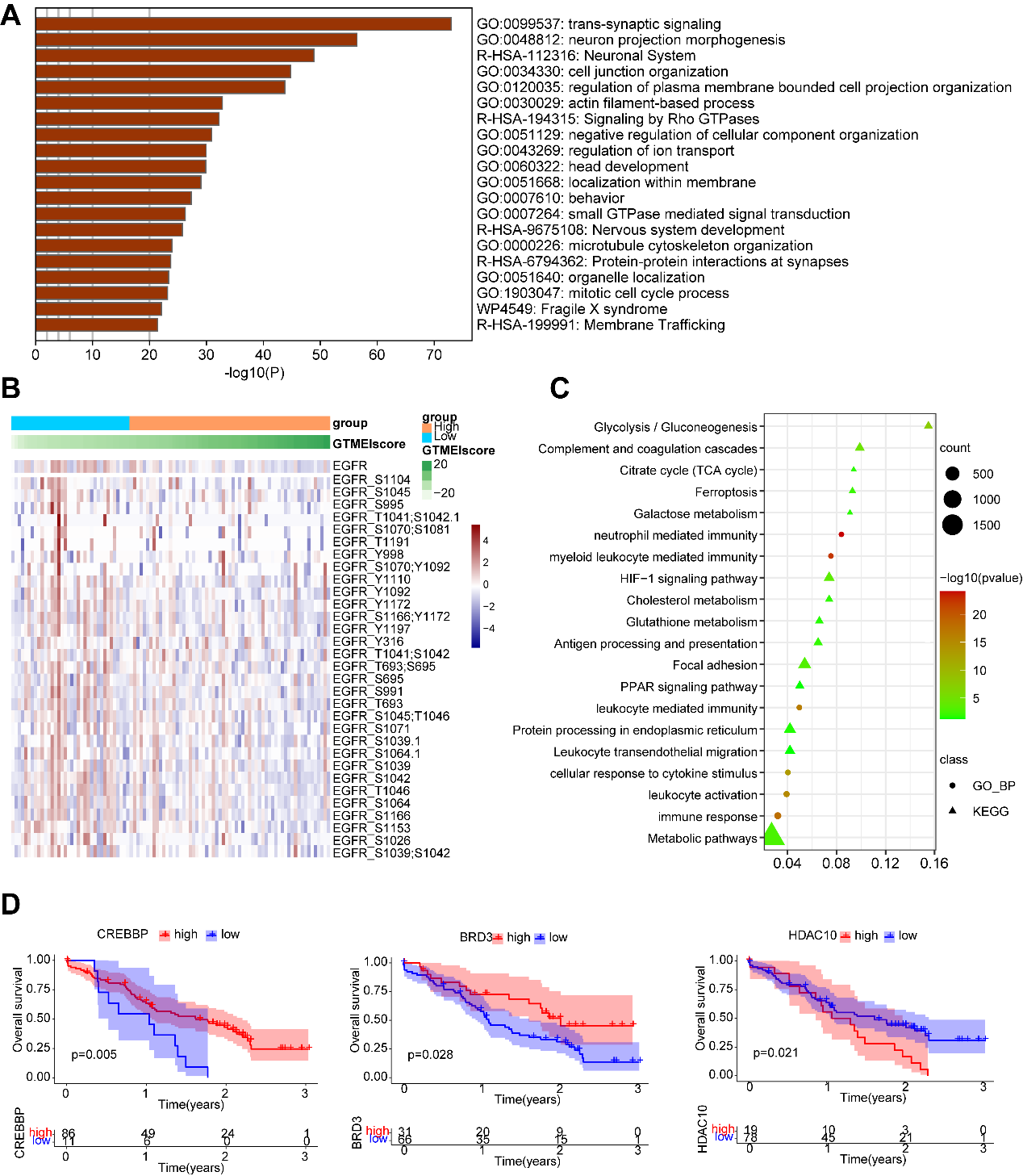


**Supplementary Figure 4 Correlation between the GTMEIscore and protein phosphorylation and acetylation. A** Bar graph of enrichment terms for proteins with significantly downregulated phosphorylation levels in the high GTMEIscore group colored by P value. **B** Heatmap showing the expression of EGFR protein and its downstream protein phosphorylation sites. **C** KEGG and GO BP enrichment analysis of proteins with significantly different acetylation levels. **D** Kaplan-Meier curves for the OS of Wang dataset GBM patients with (**left**) high CREBBP and low CREBBP expression (log-rank test P = 0.005), (**Middle**) high BRD3 and low BRD3 expression (log-rank test P = 0.028), and (**right**) high HDAC10 and low HDAC10 expression (log-rank test P = 0.021).


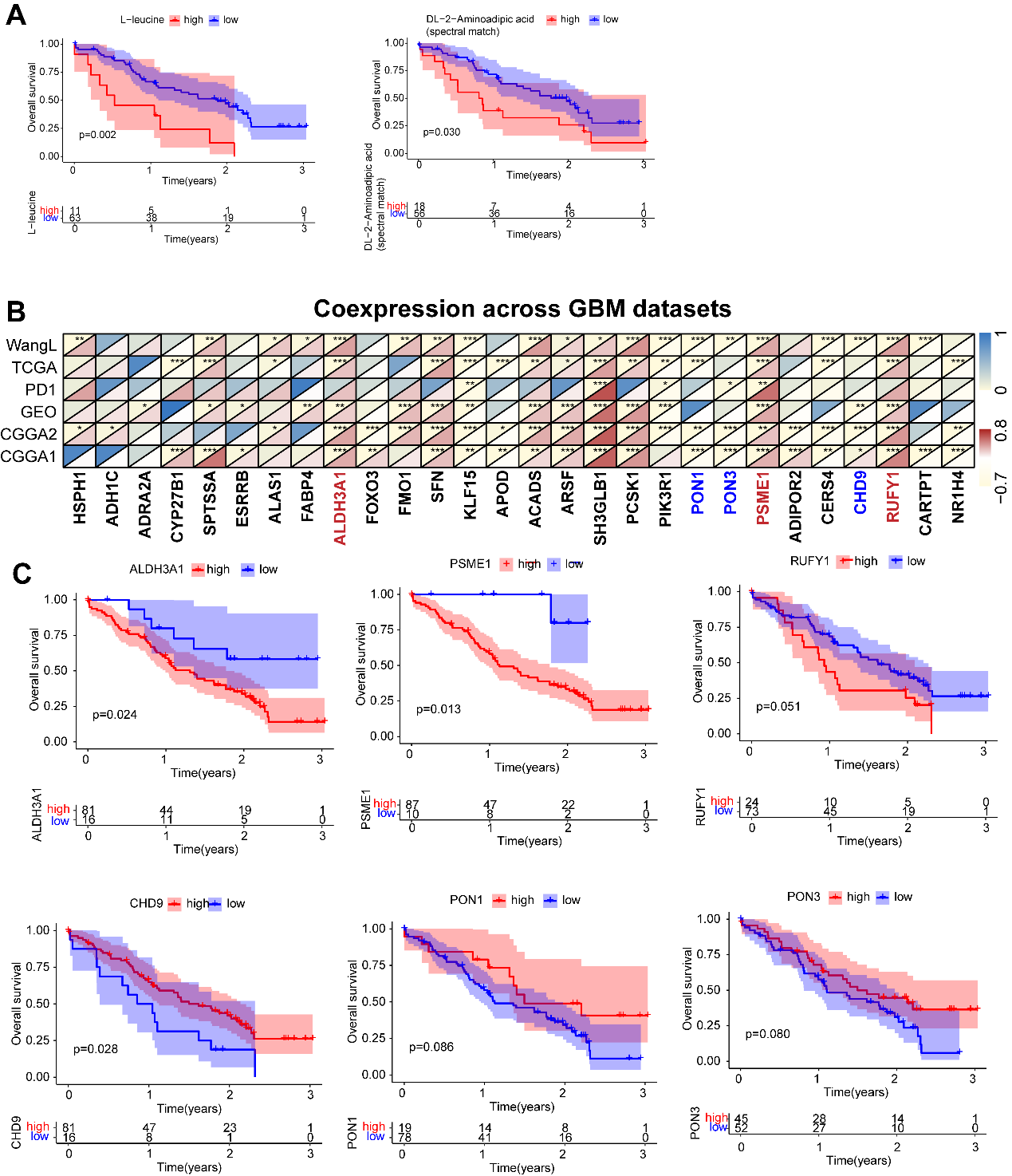


**Supplementary Figure 5 Correlation between the GTMEIscore and metabolomic and lipidomic characteristics. A** Kaplan-Meier curves for the OS of Wang dataset GBM patients with (**left**) high L-leucine and low L-leucine levels (log-rank test P = 0.002) and (**right**) high DL-2-aminoadipic acid (spectral match) and low DL-2-aminoadipic acid (spectral match) levels (log-rank test P = 0.030). **B** Spearman analysis of the correlation of the GTMEIscore with 29 metabolic regulatory genes associated with GBM prognosis. Colors indicate correlation coefficients, with yellow indicating a negative correlation and red indicating a positive correlation. Asterisks indicate statistically significant P values calculated using Spearman correlation analysis. *P < 0.05; **P < 0.01; ***P < 0.001. **C** Kaplan-Meier curves of the OS of GBM patients based on their protein levels of six genes, ALDH3A1, PSME1, RUFY1, CHD9, PON1 and PON3l, in the Wang dataset.


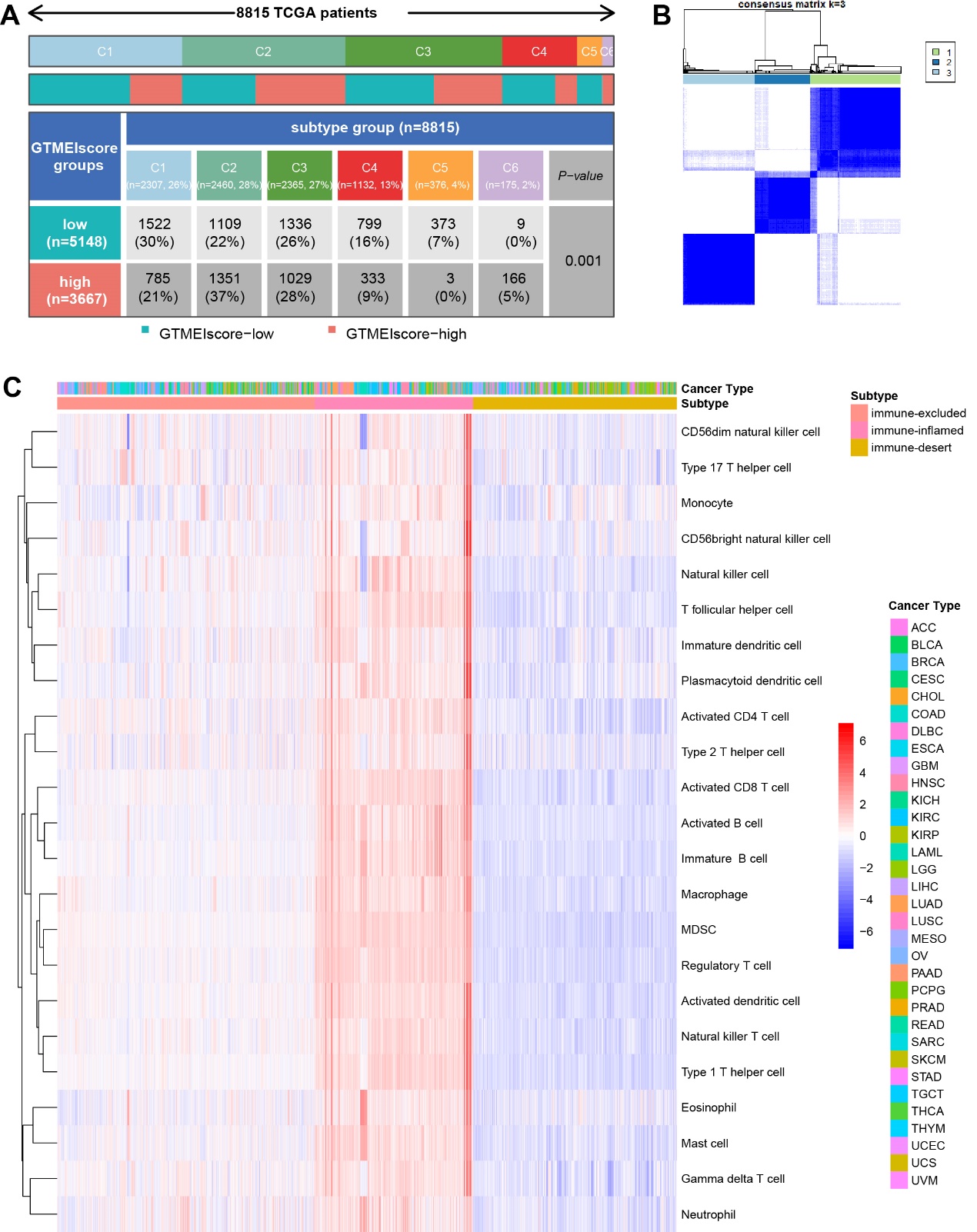


**Supplementary Figure 6 Differences in the GTMEIscore between different immune subtypes. A** Heatmap and table of the distribution of six immune molecular subtypes between the high and low GTMEIscore groups, chi-square test showed P=0.001. **B** Consensus matrix of TCGA pan-cancer samples with K=3, displaying the stability of the subtypes using 1000 iterations of hierarchical clustering. **C** Unsupervised clustering of 23 immune cell types in TCGA pan-cancer samples. A heatmap was used to visualize immune cell infiltration. Red represents high immune cell abundance, white represents moderate immune cell abundance, and blue represents low immune cell abundance.


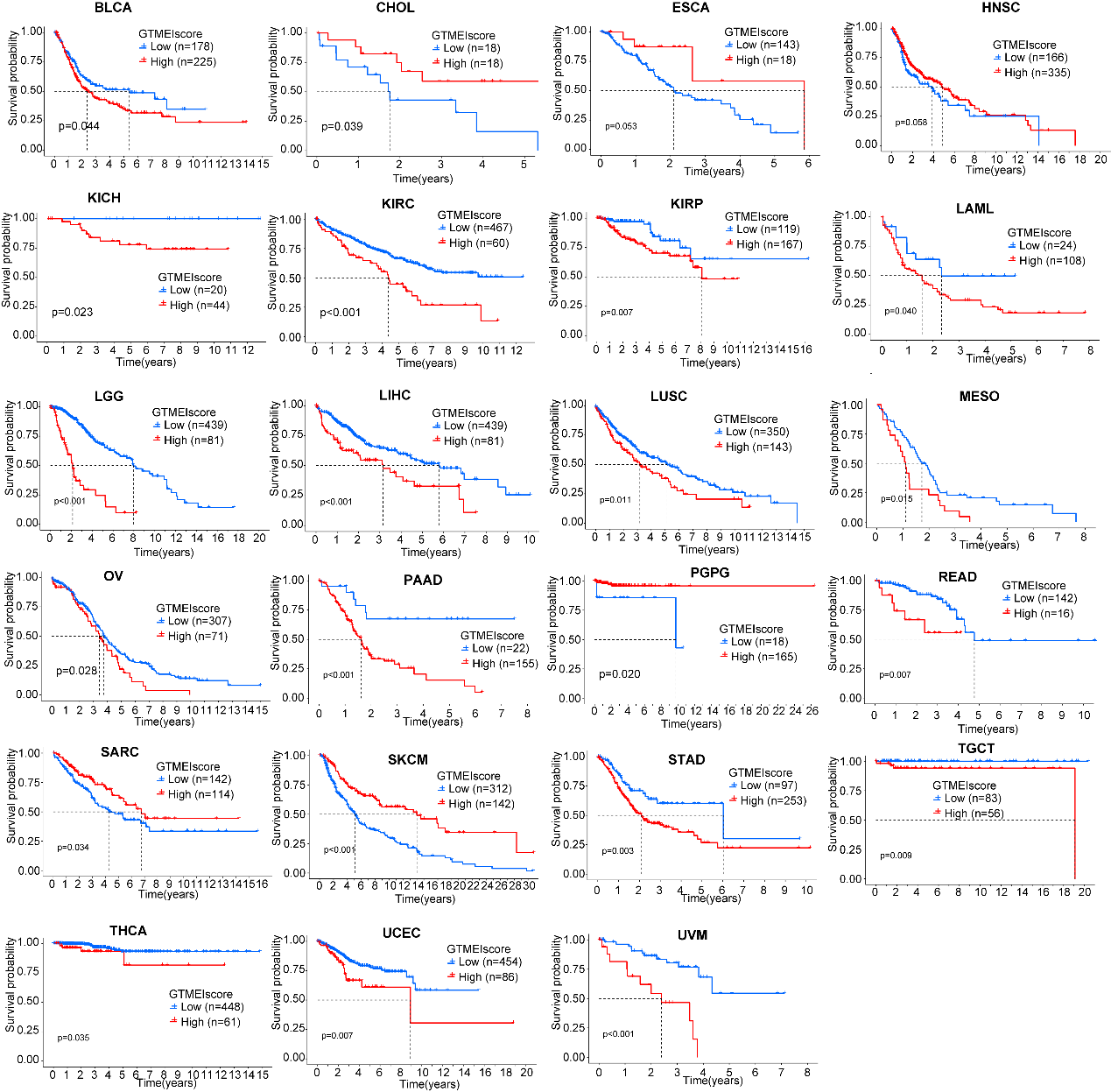


**Supplementary Figure 7 Pan-cancer and cancer-specific Kaplan–Meier survival curves of tumor patients between TISIhigh and TISIlow groups. The P-value was calculated by the log-rank test.**


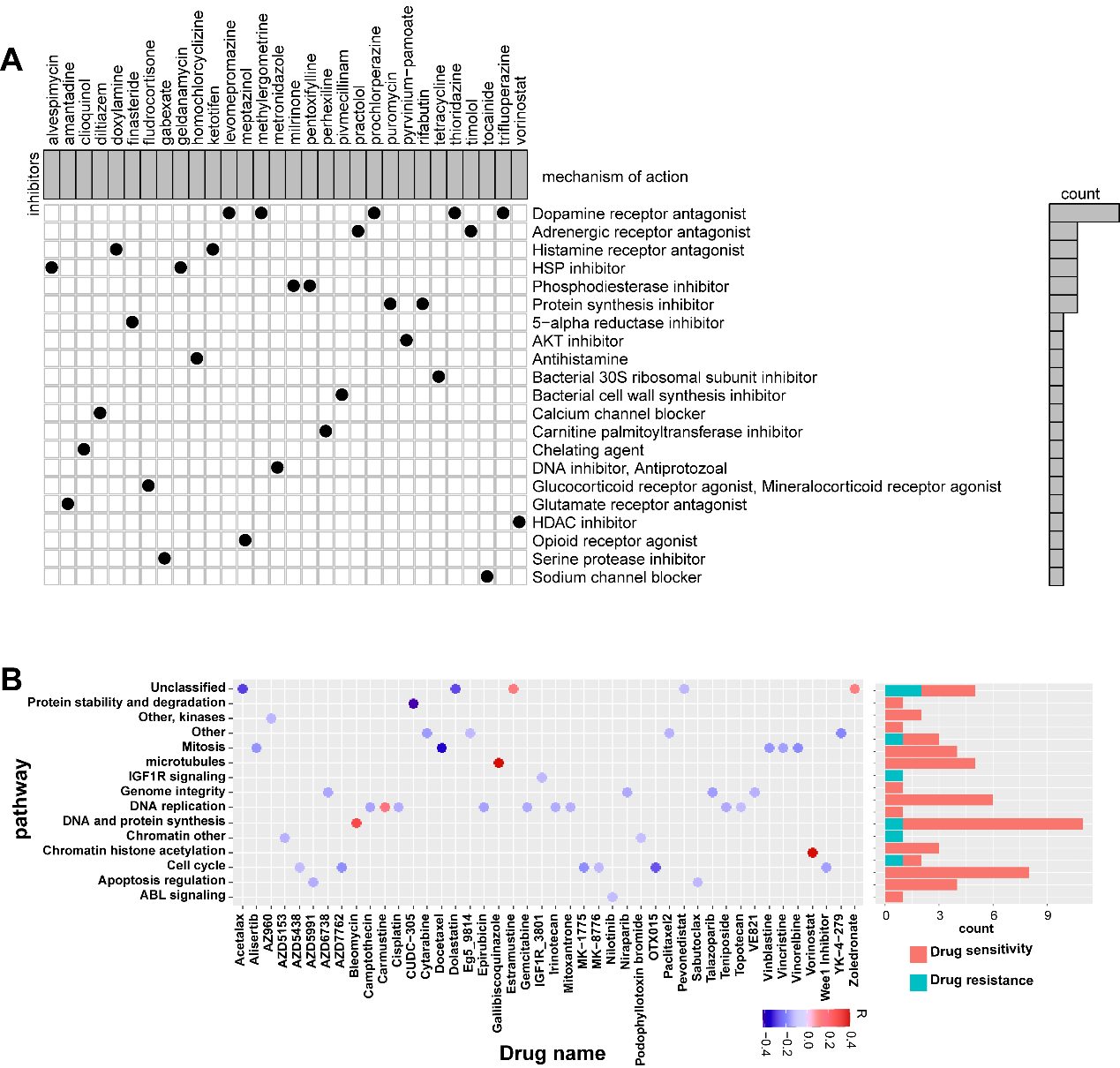


**Supplementary Figure 8 Correlation of the GTMEIscore with drug sensitivity in otner 32 cancer types. A** Heatmap showing each compound (perturbagen) from the CMap that shares mechanisms of action (rows) and sorted by descending number of compound with shared mechanisms of action.
